# The YAP1/TAZ-TEAD transcriptional network regulates gene expression at neuromuscular junctions in skeletal muscle fibers

**DOI:** 10.1093/nar/gkad1124

**Published:** 2023-12-04

**Authors:** Lea Gessler, Danyil Huraskin, Yongzhi Jian, Nane Eiber, Zhaoyong Hu, Tomasz J Prószyński, Said Hashemolhosseini

**Affiliations:** Institute of Biochemistry, Medical Faculty, Friedrich-Alexander-University of Erlangen-Nürnberg, 91054 Erlangen, Germany; Institute of Biochemistry, Medical Faculty, Friedrich-Alexander-University of Erlangen-Nürnberg, 91054 Erlangen, Germany; Institute of Biochemistry, Medical Faculty, Friedrich-Alexander-University of Erlangen-Nürnberg, 91054 Erlangen, Germany; Institute of Biochemistry, Medical Faculty, Friedrich-Alexander-University of Erlangen-Nürnberg, 91054 Erlangen, Germany; Nephrology Division, Department of Medicine, Baylor College of Medicine, Houston, TX, USA; Łukasiewicz Research Network-PORT Polish Center for Technology Development, Wrocław, Poland; Institute of Biochemistry, Medical Faculty, Friedrich-Alexander-University of Erlangen-Nürnberg, 91054 Erlangen, Germany; Muscle Research Center, Friedrich-Alexander-University of Erlangen-Nürnberg, 91054 Erlangen, Germany

## Abstract

We examined YAP1/TAZ-TEAD signaling pathway activity at neuromuscular junctions (NMJs) of skeletal muscle fibers in adult mice. Our investigations revealed that muscle-specific knockouts of *Yap1* or *Taz*, or both, demonstrate that these transcriptional coactivators regulate synaptic gene expression, the number and morphology of NMJs, and synaptic nuclei. *Yap1* or *Taz* single knockout mice display reduced grip strength, fragmentation of NMJs, and accumulation of synaptic nuclei. *Yap1*/*Taz* muscle-specific double knockout mice do not survive beyond birth and possess almost no NMJs, the few detectable show severely impaired morphology and are organized in widened endplate bands; and with motor nerve endings being mostly absent. Myogenic gene expression is significantly impaired in the denervated muscles of knockout mice. We found that *Tead1* and *Tead4* transcription rates were increased upon incubation of control primary myotubes with AGRN-conditioned medium. Reduced AGRN-dependent acetylcholine receptor clustering and synaptic gene transcription were observed in differentiated primary *Tead1* and *Tead4* knockout myotubes. *In silico* analysis of previously reported genomic occupancy sites of TEAD1/4 revealed evolutionary conserved regions of potential TEAD binding motifs in key synaptic genes, the relevance of which was functionally confirmed by reporter assays. Collectively, our data suggest a role for YAP1/TAZ-TEAD1/TEAD4 signaling, particularly through TAZ-TEAD4, in regulating synaptic gene expression and acetylcholine receptor clustering at NMJs.

## Graphical Abstract

**Graphical Abstract:**
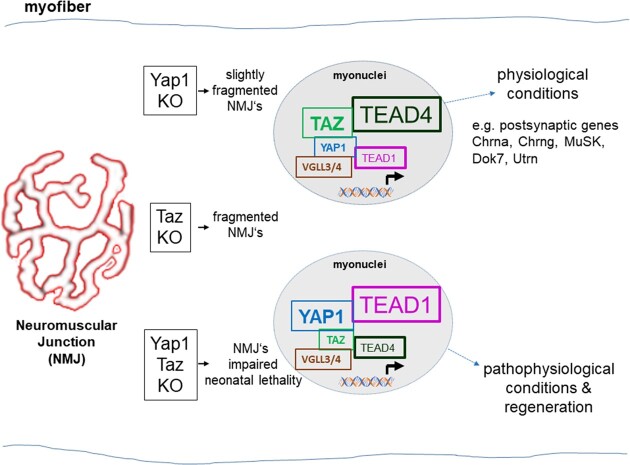

## Introduction

The Hippo pathway governs the control of organ size, tissue regeneration and stem cell self-renewal (1). In mammals, the activation of kinases MST1/2 (also known as STK4/3; homologs of Drosophila Hippo) and LATS1/2 leads to LATS-dependent phosphorylation of TAZ (transcriptional co-activator with PDZ-binding motif, also known as WWTR1) and YAP1 (Yes-associated protein, also known as YAP), thereby decreasing their stability, nuclear localization and transcriptional activity (2). Recently, YAP1 was identified as a crucial regulator of muscle fiber size (3). Furthermore, YAP1/TAZ are incorporated within the β-catenin destruction complex and subsequently regulate the Wnt response mediated via canonical Wnt signaling (4,5). YAP1 and TAZ acting as transcriptional co-activators, bind to TEA-domain transcription factor (TEAD) family members to enable the activation of transcription for TEAD target genes (6). The mammalian TEAD transcription factors consist of a family of four genes (TEAD1-4). TEAD1 was first identified as the SV40 transcriptional enhancer factor 1 (TEF-1). It binds to the specific sequence 5′-CATTCCA-3′ in SV40 Sph and GT-IIC enhancers, as well as to M-CAT motifs 5′-CATTCCT-3′ found in the promoter and regulatory regions of various muscle-specific genes (7–9). YAP1/TAZ bind TEADs at promoters, but even more at distal enhancers of target genes. At their target sites, YAP1 and TAZ cooperate with other transcriptional regulators, including AP-1, to activate target gene expression by stimulating *de novo* transcription initiation or mediating transcriptional pause release (10). Conversely, they can also inhibit transcription (1,11,12). The YAP1-TEAD complex has been crystalized (13,14) and shows a 1:1 interaction between YAP1 and TEAD. The crystal structure of TAZ-TEAD4 complex reveals two binding modes (15), one of which is similar to the YAP1-TEAD structure. The second shows two TAZ bind to two TEAD4 molecules. The formation of a TAZ-TEAD heterotetramer complex could result in a stronger additive effect if tandem TEAD binding sites are present (15). The binding partners of TEADs also include VGLL (Vestigial Like Family Member) proteins which lack a DNA-binding domain and regulate transcription by binding to TEAD transcription factors such as YAP1 and TAZ. VGLL proteins utilize their Tondu domain (16) to bind via two interfaces to the same TEAD site that YAP1 also binds to via three interfaces (13,17). VGLL1-3, which are single-Tondu-domain proteins, are expressed in specific tissues and act as transcriptional coactivators for TEAD factors (18–20). Conversely, VGLL4, which contains two Tondu domains, is expressed ubiquitously in all tissues (18) and inhibits YAP1-TEAD-mediated transcription (21).

Gene transcription mediated by transcriptional co-activators YAP1/TAZ is one of the essential regulators of muscle cell differentiation (22,23). Previous proteomic studies have shown that VGLL3 binds transcription factors TEAD1, TEAD3 and TEAD4. Meanwhile, transcriptomic analysis have indicated that VGLL3 regulates the negative feedback loop of the Hippo pathway. It affects the expression of genes that control myogenesis, including *Myf5*, *Pitx2/3*, *Wnts* and IGF-binding proteins (24). Our lab demonstrated that AXIN2 and YAP1/TAZ-TEAD signaling components are co-expressed in adult skeletal muscle fibers and canonical Wnt proteins stimulate both canonical Wnt signaling and *Axin2* expression, as well as YAP1/TAZ-TEAD signaling activity, during differentiation of muscle cells to regulate formation of myotubes (25).

At the neuromuscular junction (NMJ) various signaling pathways are responsible for clustering of nicotinic acetylcholine receptors (AChRs, CHRNs) at the postsynaptic apparatus (26). A neural isoform of a heparansulfate proteoglycan, called AGRIN (AGRN), is released by the nerve ending and participate in both the stabilization of already clustered CHRNs and stimulation of synaptic gene expression. The terms non-active and active (or neural) AGRN signify splice variants capable of clustering CHRNs. To achieve this, neural AGRN interacts with its receptor LRP4 (low density lipoprotein receptor-related protein 4), which in turn activates the co-receptor MUSK (MuSK), a muscle-specific receptor tyrosine kinase. The clustering of CHRNs serves as a hallmark for the presence of the postsynaptic apparatus within the endplate zone, the central part of each muscle fiber (26). CTNNB1 (β-catenin) is a transcriptional coactivator and the key element of the canonical Wnt signaling pathway. Muscular CTNNB1 gain-of-function phenotype is linked to presynaptic defects *in vivo* resulting from changed neuromuscular retrograde signaling (27–29). Nevertheless, *Ctnnb1* loss of function also impacts CHRN cluster size and distribution (27). In cultured muscle cells CTNNB1 exerts both positive and negative regulation of CHRN clustering. Specifically, it acts as a cytosolic link between RAPSN (RAPSYN), a peripheral membrane protein required for CHRN clustering at NMJs, and the cytoskeleton. Alternatively, CTNNB1 negatively regulates *Rapsn* expression in the nucleus (30,31). Conversely, the AXIN2-lacZ reporter mouse model indicated the activity of canonical Wnt signaling and TCF/LEF target gene expression at the NMJ, as evident from the β-galactosidase reporter accumulation in synaptic nuclei in muscle fibers (25). Similarly, in X-Gal stained TCF/LEF-lacZ reporter mouse muscles a pronounced neuromuscular signal is detectable (32). YAP1 has been found to accumulate at the NMJs, with increased expression upon denervation of the muscle, to counteract neurogenic muscle atrophy (3). In a prior study, deletion of *Yap1* in muscle resulted in smaller and more widely distributed CHRN clusters, which were not fully covered by nerve terminals leading to functional deficits and reduced reinnervation after denervation (33). Mechanistically, reduced CTNNB1 activity downstream of YAP1 in *Yap1* knockout muscles is the underlying mechanism, which could be partly restored using LiCl treatment (33).

As our understanding of the neuromuscular role of YAP1/TAZ-TEAD signaling develops, the specific involvement of transcriptional co-activators YAP1 and TAZ continues to remain elusive. Research investigating NMJs of muscle-specific *Yap1* mutant mice indicates that muscle YAP1 plays a role in the size and subcellular localization of CHRN clusters (33). It is unclear which of the four TEAD transcription factors are activated by YAP1 and/or TAZ to induce transcription of target genes involved in muscle fiber biology. In the present study we set out to identify the transcriptional effectors of YAP1/TAZ-TEAD signaling, which are expressed in differentiated muscle cells to regulate synaptic gene expression to maintain physiological function, occurrence, and morphology of NMJs.

## Materials and methods

### Plasmids, mutagenesis, primers, *in situ* probes

To generate the luciferase constructs the respective genomic regions were amplified with primers containing KpnI or XhoI restriction digestion sites (Supplementary Table S1) for directional insertion into the pGL4.20-Hsp68min vector (a gift from Michael Wegner, FAU Erlangen-Nürnberg, Germany), which is a pGL4.20[luc2/Puro] vector (Promega, E675A) modified by insertion of the Hsp68 minimal promoter sequence. M-CAT motifs within genomic regions were mutagenized in analogy to previous M-CAT motif mutagenesis approaches (34,35) by using the Q5 Site-Directed Mutagenesis Kit (New England Biolabs, E0554S). The mutagenesis primers designed and used are listed in the Supplementary Table S1. Always correct clones were verified by restriction digestion and/or DNA sequencing.

To generate the pcDNA3.1–6xHA-Tead1 and pcDNA3.1–6xHA-Tead4 expression plasmids mouse full length Tead1 and Tead4 cDNA sequences were amplified from mouse muscle 1^st^-strand cDNA with primers as listed (Supplementary Table S1), then ligated into a linearized pcDNA3.1–6xHA vector (36), which encodes the respective protein tagged amino-terminally with six tandem copies of the HA-tag. After plasmid verification by restriction digestion and sequencing, expression of the proteins was assessed by transfection of constructs at different dilutions into HEK293 cells and detection of proteins by specific antibodies targeting the respective proteins and the HA-tag.

For generation of *in situ* riboprobes, corresponding regions were amplified from mouse muscle 1^st^-strand cDNA using the same primers as for the quantification of respective transcripts in qPCR studies (Supplementary Table S1) and ligated into EcoRV digested pBluescript SK(+) plasmid. Directionality and correct sequence of the insert was verified by DNA sequencing. Riboprobes were made by linearization of the plasmid and transcription with the T7 RNA polymerase.

pCMV-flag YAP2 5SA encoding constitutive active mutant of YAP1 (37) and pEF-TAZ-N-Flag (S89A) vector encoding constitutive active mutant of TAZ (38) were acquired from Björn von Eyss (Fritz-Lipmann-Institut, Jena, Germany). pMAX-GFP (Lonza, VPD-1001) was used as control for transfection.

### *in situ* hybridization, RNA extraction, reverse transcription, PCR

For *in situ* hybridization experiments, newborn wild type pups were decapitated immediately after birth and diaphragm was dissected and fixed overnight in 4% paraformaldehyde (PFA) and dehydrated in gradient of 25% to 100% methanol solutions for 15 min each. Diaphragms were stored at –20°C. To perform *in situ* hybridization diaphragms were rehydrated, quickly washed in PBST and treated for 15 min with Proteinase K (20 μg/ml). After refixation in 0.2% glutaraldehyde in 4% PFA, diaphragms were washed, incubated for 2 h in pre-hybridization buffer and hybridized overnight at 55°C with corresponding denatured (5 min 95°C, followed by 3 min on ice) riboprobes (10 μl/ml). Next day diaphragms were washed, blocked with 10% FCS in TBST and incubated for 4 h with a 1:2000 dilution of the anti-Digoxigenin-AP antibody (Roche Diagnostics, 11093274910) in 1% FCS in TBST. After six washing steps, diaphragms were kept in TBST overnight. Next day diaphragms were equilibrated in NTM solution (0.1 M Tris, pH 9.5, 0.5 M NaCl, 0.05 M MgCl2, 0.1% Tween 20) and developed with 90 mM NBT (Roche Diagnostics, 11383213001) and 110 mM BCIP (Roche Diagnostics, 11383221001) in NTM solution.

Total RNA was extracted from primary muscle cells, hind limb muscle of newborns, or the extensor digitorum longus/gastrocnemius muscle of adult mice with TRIzol reagent (Thermo Fisher Scientific, 15596026) (39) and reverse transcribed with M-MuLV Reverse Transcriptase (New England Biolabs, M0253) according to the manufacturer's instructions. cDNAs were used with mouse-specific primers (Supplementary Table S1) for quantitative PCR reactions using the PowerUp SYBR Green Master Mix (Thermo Fisher Scientific, A25743) and the C1000 Thermal Cycler with the CFX96 Real-Time PCR Detection System (Bio-Rad) according to the manufacturer's instructions. After the PCR run, sizes of amplified DNA products were verified by agarose gel electrophoresis. Ct values of the genes of interest were normalized to Ct values of the internal control (Rpl8 gene) and related to the control sample (fold change = 2^−ΔΔCT^) (40,41).

### Tissue culture, culturing of primary muscle cells, transfection

Primary skeletal muscle satellite cells were prepared from muscles of ∼3–6 months old adult C57BL/6 wild type, control or knockout mice using the mouse Skeletal Muscle Dissociation Kit (Miltenyi Biotech, 130–098-305), followed by mouse MACS Satellite Cell Isolation Kit (Miltenyi Biotech, 130-104-268). Cells were used for immediate RNA extraction or seeded on Matrigel-coated plates (Thermo Fisher Scientific, CB-40234) in growth medium (40% DMEM, 40% Ham's F10, 20% FCS, 1% penicillin/streptomycin, and recombinant human fibroblast growth factor (Promega, G507A, 5 ng/ml)). To yield sufficient total RNA amounts from directly isolated muscle satellite cells for cDNA synthesis, a total of eight grams of mouse muscle tissue was used for isolation and the cells were pooled before RNA extraction. For differentiation to myotubes, primary skeletal muscle cells were grown to confluency and cultured in differentiation medium (95% DMEM, 5% horse serum, 1% penicillin/streptomycin).

To generate cultured Yap1/Taz knockout myotubes, satellite cells were extracted from Yap1/Taz^loxP/loxP^::Pax7-CreER^T2^::R26R^YFP/+^ mice, incubated after 6 days for 2 days with 4-hydroxy tamoxifen (Sigma Aldrich, 10nM, H7904), and differentiated for 8 days to myotubes on laminin-coated plates.

Cultured primary skeletal muscle cells were transfected by nucleofection with the Amaxa NHDF Nucleofector Kit (Lonza, VPD-1001), cultured C2C12 myoblasts with the Amaxa Cell Line Nucleofector Kit V (Lonza, VCA-1003) in the Nucleofector 2b Device (Lonza, AAB-1001) according to the manufacturer's instructions. Each sample was transfected with 4μg of plasmid DNA. To control for transfection efficiency 0.2μg of the supplied pMAX-GFP was added to each sample's DNA, and GFP expression was verified 24h after transfection with the Leica DM IRB microscope.

To induce clustering of CHRNs, cells were seeded onto 0.1% gelatine (Thermo Fisher Scientific, Cascade Biologics Attachment Factor 1x, S-006–100) coated dishes, differentiated to myotubes for 6 days and treated with neural AGRN-conditioned media. The production of AGRN-conditioned media was described previously (42). AGRN-conditioned medium was added at 1:8 dilution to myotubes. CHRN clusters were detected and quantified 16 h later, as described below.

### Protein lysates, SDS-PAGE, western blot

For preparation of muscle tissue extract, synaptic areas of mouse gastrocnemius or tibialis anterior muscles were dissected, frozen in liquid nitrogen, mashed, and homogenized in ice cold lysis buffer (10 mM HEPES at pH 7.9, 0.2 mM EDTA, 2 mM DTT, 1% Nonidet *P*-40, 2 μg/μl Leupeptin and Aprotinin) for 10 min (88).

To obtain cytosolic and nuclear fractions of protein, cells were lysed in ice cold protein lysis buffer A (10 mM HEPES pH 7.9, 10 mM KCl, 0.2 mM EDTA, 2 mM DTT, 20 μg/ml Aprotinin and 20 μg/ml Leupeptin in deionized water) and scraped off with a cell scraper into a reaction tube. After 5 min on ice, 1% NP-40 was added and vortexed for 10 s. The lysate was centrifuged for 30 s at 16 000×g to acquire the supernatant containing the cytosolic protein fraction. The pellet was resuspended in the same lysis buffer with additional 400 mM NaCl and 1% NP-40 and rotated for 15 min at 4°C before centrifugation for 5 min at 16 000×g to acquire the nuclear protein fraction in the supernatant. Whole cell extracts were prepared by scraping the cells into protein lysis buffer A, incubating on ice for 5 min and addition of 1% NP-40 before vortexing for 10 s. Then 400 mM NaCl was added and the lysate was rotated for 15 min at 4°C. After centrifugation for 5 min at 16 000×g the supernatant containing the whole cell protein extract was used. All protein lysates were diluted with Laemmli buffer, boiled at 95°C for 5 min, and separated by sodium dodecyl sulfate (SDS) polyacrylamide gel electrophoresis with the Biometra Minigel Twin system. Separated proteins were blotted on to a nitrocellulose membrane (Sigma Aldrich, Protran BA 85), blocked in 5% BSA or 5% non-fat dry milk in PBS or TBS with 0.1% Tween20 slowly shaking for 1 h at room temperature.

After blocking the membranes were incubated with primary antibodies at 1:3000 dilution slowly shaking over night at 4°C: TEAD1 (Cell Signaling, 12292), TEAD4 (Abnova, H00007004-M01), YAP1/TAZ (Cell Signaling, 8418), YAP1 63.7 (dilution 1:2000, Santa Cruz Biotechnology, sc-101199), TAZ 1F1 (dilution 1:2000, Santa Cruz Biotechnology, sc-293183), Phospho-YAP1 (Ser127) (Cell Signaling, 13008), GAPDH (Santa Cruz Biotechnology, sc-25778), MUSK 20kd (dilution 1:100, (39)), DOK7 (dilution 1:1000, Santa Cruz Biotechnology, sc-50464), MYOG (dilution 1:1000, DSHB, AB_2146602), CHRNA1 (dilution 1:100, DSHB, AB_528405). Corresponding HRP-linked secondary antibodies against rabbit, mouse, or rat (Cell Signaling, 7074, 7076, 7070) at a 1:3000 dilution were bound for 2 h at room temperature. Protein bands were detected with chemiluminescence reagent solution and protein bands were exposed on RXSuper X-ray films (Fuji Medical). The chemiluminescence reagent consisted of 3 ml of 0.25 mg/ml Luminol (Sigma Aldrich, A-4685) in 0.1M Tris pH 8.6 solution and 40μl of 1.1 mg/ml Para-hydroxy-cumarinic acid (Sigma Aldrich, C-9008) in DMSO, mixed with 3 ml of 1× PBS and 1.2 μl of 30% H_2_O_2_. For difficult-to-detect proteins, Amersham ECL Advance Western Blotting Detection Kit was used instead (GE Healthcare, RPN2135) according to manufacturer's instructions. Western blot results were quantified by densitometric analysis using Fiji image processing package (https://fiji.sc/) (43). Films were scanned with an Epson Expression 1600 Pro Scanner at 600 dpi. After background subtraction, protein bands of interest were labeled and measured. For quantification the protein band intensity was normalized to the intensity of the GAPDH protein band of the respective sample.

### Quantitative 2D and 3D Morphometrical Imaging, Immunofluorescence staining, Fluorescence Microscopy

For immunofluorescence experiments, newborn pups were decapitated immediately after birth and diaphragm was dissected and fixed overnight in 2% PFA. After washing three times 30 min each with 1xPBS, the diaphragm was blocked in 100 mM glycine for 30 min at room temperature. Next the diaphragm was incubated with rhodamine-congujated α-bungarotoxin (BTX, 1:2500, Invitrogen) for 30 min at room temperature. The primary antibodies rabbit anti-Neurofilament (1:1000, SIGMA, N4142) and rabbit anti-Synaptophysin (1:1000, SIGMA, SAB4502906) were applied in antibody dilution buffer (0.5 M NaCl, 0.01M phosphate buffer, 1% BSA, 0.3% Triton-X-100) for four nights. After washing three times 30 min with 0.5% Triton X-100, the secondary antibody conjugated to Alexa-488 (gt-anti rabbit Alexa488, 1:1000, Thermo Fisher A-11034) was applied. Stainings were documented using a Zeiss Axio Examiner Z1 microscope (Carl Zeiss MicroImaging) equipped with an AxioCam MRm camera (Carl Zeiss MicroImaging) and ZEISS AxioVision Release 4.8 (Carl Zeiss MicroImaging) (44). The endplate band width was determined with LSM 5 Image Examiner (Carl Zeiss MicroImaging).

For immunofluorescence analysis cells were fixed in 2% PFA for 15 min on ice, permeabilized for 10 min in 0.1% Triton X-100 in PBS, blocked in 10%FCS (v/v), 1%BSA (v/v) in PBS for 1 hour at room temperature and incubated with antibodies at 1:1000 dilution at 4°C overnight. Secondary antibodies conjugated to Cy3 or Alexa Fluor 488 immunofluorescent dyes (Dianova, 111-165-144, 115-165-146) were used for detection.

For quantitative 2D and 3D morphometrical imaging, mouse soleus muscle was dissected and fixed in 2% PFA for 2 h at 4°C. For detection of CHRNs, muscle bundles containing 5–10 fibers were prepared and stained with BTX (1:2.500, Invitrogen), and primary antibodies rabbit anti-Neurofilament (1:1000, SIGMA, N4142) and rabbit anti-Synaptophysin (1:1000, SIGMA, SAB4502906), for 1 h at room temperature. Stained bundles were washed three times 5 min in phosphate buffered saline (PBS) and embedded in Mowiol. Then, 3D images of NMJs were taken with a 63× oil objective (Zeiss Examiner E1, Carl Zeiss MicroImaging). Images were deconvoluted and analyzed using different modules in AxioVision Software (ZEISS AxioVision Release 4.8, Carl Zeiss MicroImaging). Volume, surface area, and the number of fragments were determined for each NMJ. For each genotype, more than 50 NMJs were analyzed (44). For 2D imaging, aNMJ-morph, an ImageJ software based platform (45) was used to analyse NMJ morphology (46).

For detection of CHRNs in cells, counterstaining was performed with DAPI to visualize nuclei. To quantify CHRN clusters images of BTX and DAPI stained myotubes were acquired with a 20x objective on a Leica DMI6000B microscope (Leica Microsystems), exported as TIF image files and quantified with Fiji software. A constant threshold was set for all samples to subtract background signal and create a mask for quantification of BTX fluorescence signal intensity with the Analyze Particles function. The normalized BTX fluorescence intensity was calculated as the total raw integrated density of BTX fluorescence signal divided by the number of nuclei in the image. Signals from undifferentiated cells or cell debris were excluded prior to quantification by manual selection.

### RNA-seq, in silico analysis of putative Tead binding sites

For RNA-seq comparisons of innervated versus denervated hind limb gastrocnemius muscles from male mice the NIH GEO dataset with the accession number GSE217577 was used. For *in silico* analysis of putative TEAD binding sites in synaptic genes, ChIP-Seq data of TEAD4 and TEAD1 occupation in C2C12 cells at day 0 and day 6 of differentiation (47) were screened for occupation in regions overlapping with Chrna1, Musk and Dok7 genes and confined by their respective 5′ and 3′ neighboring genes. First, the UCSC Genome Browser (https://genome-euro.ucsc.edu/index.html) (48) Mouse July 2007 (NCBI37/mm9) Assembly was searched for the gene of interest and used to identify the respective chromosome and base positions. Then the Supplementary S1 dataset from (47) was searched manually for TEAD1 and TEAD4 genomic occupancy sites that overlapped with these regions, all found sites are listed in (Supplementary Table S2). The genomic sequences of the respective regions were exported in FASTA format from the UCSC Browser and screened in JASPAR 2018 database (http://jaspar.genereg.net/) (49) with the basic sequence analysis ‘Scan’ tool and the TEAD4 matrix model (matrix profile MA0809.1) applied at the recommended default relative profile score threshold of 80%. The output contained a list of scored predicted TEAD4 binding sequences with their relative start and end coordinates that were used to calculate their respective chromosomal coordinates. Predicted binding sites (Supplementary Table S3) showed high basewise conservation among mammalian species in evolutionary conserved regions, as determined by positive PhyloP basewise conservation score (50). For further analysis, we considered the following genomic regions: Chrna1_6–7 (one predicted site), Chrna1_10–11 (two predicted sites), MuSK_170–171 (three predicted sites) and Dok7_137–138 (two predicted sites).

### Luciferase assays

Luciferase reporter assays using constructs containing genomic regions with putative TEAD binding sites were performed on C2C12 muscle cell line transfected with the respective luciferase reporter constructs or empty pGL4.20-Hsp68min plasmid (control) together with expression plasmids encoding constitutive active YAP5SA, constitutive active TAZS89A, HA-tagged TEAD1 and HA-tagged TEAD4. After transfection, cells were harvested after 48h to analyze myoblasts or differentiated to myotubes and harvested after 5 days. Cell extracts were prepared with the harvest buffer (88 mM morpholine-4-ethanesulfonic acid pH 7.8, 88 mM Tris pH 7.8, 12.5 mM Magnesium Acetate, 2.5 mM ATP, 1 mM DTT and 0.1% TritonX-100) and measured with the Berthold CENTRO LB960 luminometer. GTIIC-luciferase reporter assays were performed as described before (25).

### Mouse procedures and genotyping, surgical procedures

Mouse experiments were performed in accordance with animal welfare laws and approved by the responsible local committees (animal protection officer, Sachgebiet Tierschutzangelegenheiten, FAU Erlangen-Nürnberg, AZ: I/39/EE006 and TS-07/11), government bodies (Regierung von Unterfranken), the Wrocław Local Ethical Committee for Animal Experimentation (permission number 045/2023). *Yap1*/*Taz* floxed mice were purchased from The Jackson Laboratory (#030532) (51). Cre reporter mice were described before (52,53). Mice were housed in cages that were maintained in a room with temperature 22 ± 1°C and relative humidity 50–60% on a 12-h light/dark cycle. Water and food were provided ad libitum. Mouse mating and genotyping were performed as previously described (54). Muscle force of the mice was measured with all four limbs by a Grip Strength Test Meter (Bioseb) (44). All adult muscles which were analyzed in this manuscript commonly belong to animals of 3–6 months of age. Denervation experiments were essentially performed as described before (39). For surgery using standard aseptic techniques, animals were anesthetized and treated for pain relief by intraperitoneal administration of a ketamine-rompune mixture (90 mg/kg body weight ketamine [Pfizer], 7.5 mg/kg body weight ksylasine [Bayer], 2 mg/kg meloxicam). The wound was treated with 2% lidocaine. Treatment after surgery consisted of single daily injections of meloxicam for three days and three injections of buprenorphine for one day. A skin incision was made on the lateral thigh to expose the left biceps femoris muscle, and a longitudinal incision was made to expose and transect the sciatic nerve at the level of its trifurcation. Animals were stitched and sacrificed after 5 days post-operatively, and their soleus and gastrocnemius muscle were dissected.

### Nerve muscle preparation and extracellular recordings

Diaphragm-phrenic nerve preparations were maintained *ex vivo* in Liley's solution gassed with 95% O_2_, 5% CO_2_ at room temperature (55). The recording chamber had a volume of approximately 1 ml and was perfused at a rate of 1 ml/min. The nerve was drawn up into a suction electrode for stimulation with pulses of 0.1 ms duration. The preparation was placed on the stage of a Zeiss Axio Examiner Z1 microscope (Carl Zeiss MicroImaging) fitted with incident light fluorescence illumination with filters for 547 nm/red (Zeiss filter set 20) fluorescing fluorophore (Carl Zeiss MicroImaging). At the beginning of the experiment, the compound muscle action potential (CMAP) was recorded using a micropipette with a tip diameter of approximately 10 μm filled with bathing solution. The electrode was positioned so that the latency of the major negative peak was minimized. The electrode was then positioned 100 μm above the surface of the muscle, and CMAP was recorded. For recordings in the presence of d-tubocurarine, the chamber was filled with 2 ml (300, 800 or 1000 nM) of d-tubocurarine chloride (Sigma Aldrich). During the curare treatment, trains of 25 repetitive nerve stimulations (5 Hz) were performed at 2 min intervals, and the ratio of CMAP amplitudes (mean (20th–25th)/2nd) was calculated (44,56).

### Intracellular recordings and data analysis

To block muscle action potentials so that EPPs (endplate potentials) and EPCs (endplate currents) could be recorded (57,58), μ-conotoxin GIIIB (μ-CTX, 2 μM; Peptide Institute) was added to Lilly's solution. Concurrently, clustered CHRNs at NMJs were labeled by adding 0.5 × 10^−8^ M of BTX to the same Lilly solution. In some experiments, the effect of the toxin wore off after 1–2 h, and contractions resumed in response to nerve stimulation. These preparations were then exposed a second time to the toxin. Two intracellular electrodes (resistance 10–15 MΩ) were inserted within 50 μm of the NMJs under visual inspection (58). Current was passed through one electrode to maintain the membrane potential within 2 mV of –75 mV, while voltage transients were recorded with the other. Signals were amplified by an Axoclamp 900 A and digitized at 40 kHz by a Digidata 1440 A under the control of pCLAMP 10 (Molecular Devices). Voltage records were filtered at 3 kHz and current records at 1 kHz (8-pole Bessel filter). Current transients were recorded using the two-electrode voltage-clamp facility of the Axoclamp 900 A. Clamp gains were usually 300–1000, reducing the voltage transients to < 3% of their unclamped amplitudes. At most NMJs, 50–100 spontaneous quantal events were recorded during a period of 1 min. Records were analyzed using pCLAMP 10. Spontaneous events were extracted using the ‘template search’ facility and edited by eye to remove obvious artifacts. Events recorded from each NMJ were averaged, and the amplitude and frequency were determined (44).

### Statistical analysis

Statistical analysis was performed in GraphPad Prism 10 Software as indicated. Outliers were identified by GraphPad Prism and not used for analysis. Wherever not differently stated, unpaired student t test and SD error bars were used. *P* value Format: GraphPad style which reports four digits after the decimal point with a leading zero: ns (not significant) *P* > 0.05, * *P* ≤ 0.05, ** *P* ≤ 0.01, *** *P* ≤ 0.001, **** *P* ≤ 0.0001.

## Results

### Nerve-dependent changes of transcript amounts of YAP1/TAZ-TEAD members in skeletal muscles

**Figure 1.**
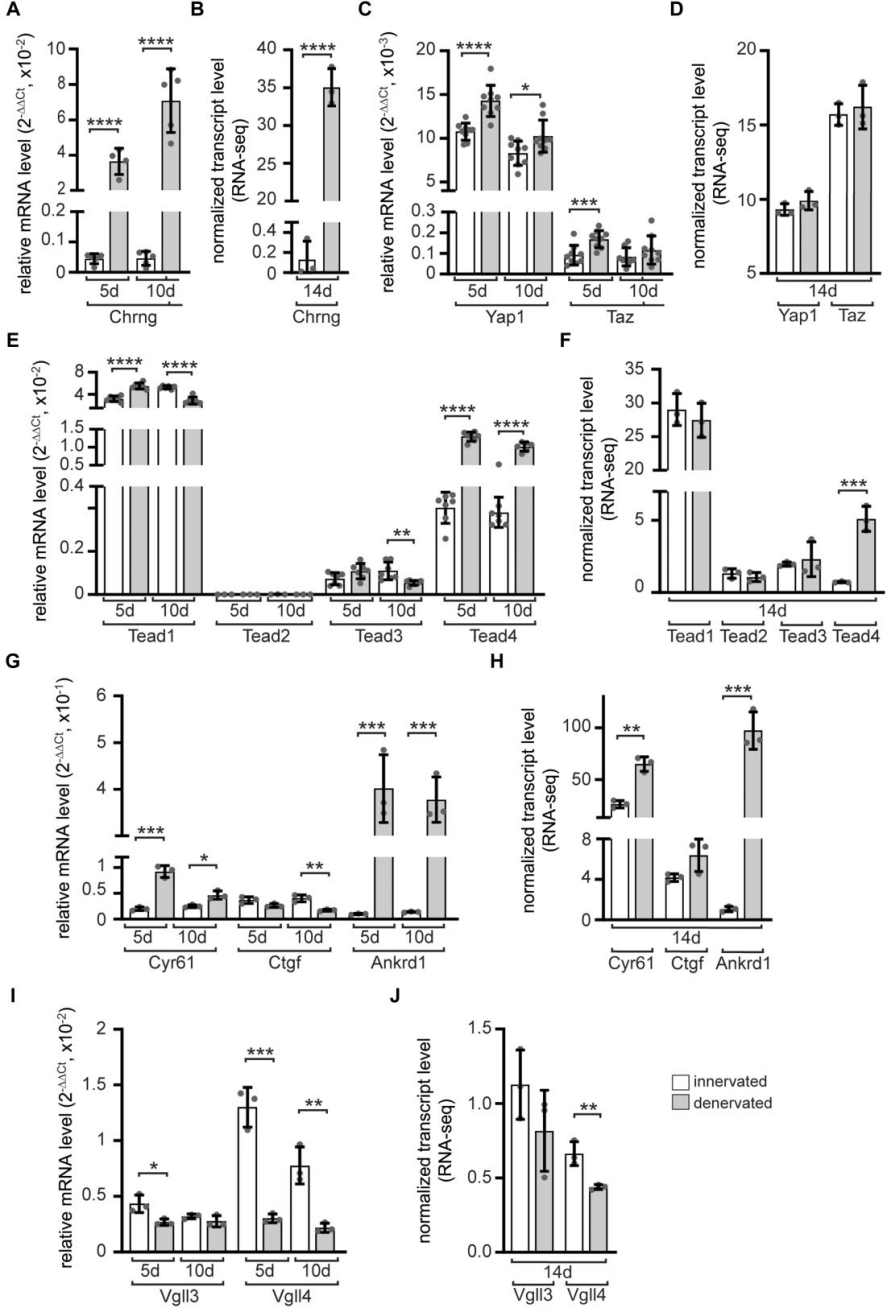
Transcriptional changes in denervated muscle were investigated for members of the YAP1/TAZ-TEAD signaling pathway. The mRNA levels of nuclear effectors of the Hippo signaling in gastrocnemius muscles denervated for 5 or 10 days were measured. The same candidates were assessed by RNA-seq after 14 days of denervation in gastrocnemius muscles. All findings are presented as graphs. Asterisks above the bars indicate the statistical significance level for transcript levels between innervated and denervated muscles. (**A, B**) The increase of *Chrng* provides significant evidence of successful denervation of the studied muscles. (**C, D**) In denervated muscle tissue, there was a notable increase in transcript levels of *Yap1* and *Taz*. (**E, F**) In response to denervation, *Tead1* and *Tead4* mRNA showed significant changes, while transcript levels of *Tead3* decreased at 10d after denervation. (**G, H**) The expression of TEAD target genes were affected by denervation. *Cyr61* and *Ankrd1* expression exhibited a significant increase, whereas *Ctgf* expression showed differential changes by qPCR and RNA-seq. (**I, J**) The transcript levels of TEAD repressors *Vgll3* and *Vgll4* were dissimilarly affected, with *Vgll3* remaining largely unchanged, while *Vgll4* transcript levels significantly decreased. qPCR was performed at least three times in duplicate for *N* ≥ 3 mice. Color coding legend of the columns within the diagrams as in (J).

Although the physical presence of nerve endings at NMJs is not required for postsynaptic gene expression (59), apparently postsynaptic gene transcription is mediated by specific signaling pathways at NMJs. For example, the ectopic expression of AGRN, neural active MUSK or NRG1/ERBB signaling upregulates the transcription of synaptic genes (60–64). The continuous presence of the nerve is not required for inducing NMJ-specific transcription but rather important for the repression of CHRN transcription outside the NMJ area. Consequently, we were interested in how denervation of the muscle would affect the transcription of *Yap1*/*Taz-Tead* signaling members. Previously, *Yap1* expression was found elevated following skeletal muscle denervation (3). We thus assessed by qPCR if and how expression of transcriptional effectors of YAP1/TAZ-TEAD signaling were changed *in vivo* in denervated gastrocnemius muscles 5 or 10 days after sciatic nerve lesion compared to contra-lateral innervated muscles of the same mouse. These qPCR data were compared with RNA-seq data of denervated gastrocnemius muscle for 14 days. Both qPCR and RNA-seq data showed mostly the same trend. Molecularly, denervation was confirmed by upregulation of the denervation hallmark of mature NMJs, namely re-expression of embryonic *Chrng* (Figure 1A, B). Expression of *Yap1* was significantly increased in denervated muscle consistent with a previous report (3), while *Taz* levels were upregulated significantly or tendencially (Figure 1C, D). Denervated muscle featured elevated expression of *Tead1* and, more prominently, *Tead4* after 5 days denervation (Figure 1E), but only a significant *Tead4* increase was confirmed by RNA-seq (Figure 1F). At all denervation time points, the expression of typical TEAD target genes was changed, *Cyr61* and *Ankrd1* were strongly upregulated (Figure 1G, H), while *Ctgf* transcripts levels were ambivalent comparing qPCR with RNA-seq data (Figure 1G, H). VGLL proteins are antagonizing YAP1/TAZ-TEAD interaction and it is of interest how their expression responds to denervation. A very prominent downregulation of *Vgll4*, and partially *Vgll3*, was detected in denervated versus innervated gastrocnemius muscle (Figure 1I, J). Overall, these data suggest that after denervation there is a shift towards stimulation of YAP1/TAZ-TEAD dependent transcriptional activity.

### Muscle strength is decreased in muscle-specific Yap1 and Taz knockout mice, while double muscle-specific Yap1 and Taz knockout mice die at birth

**Figure 2.**
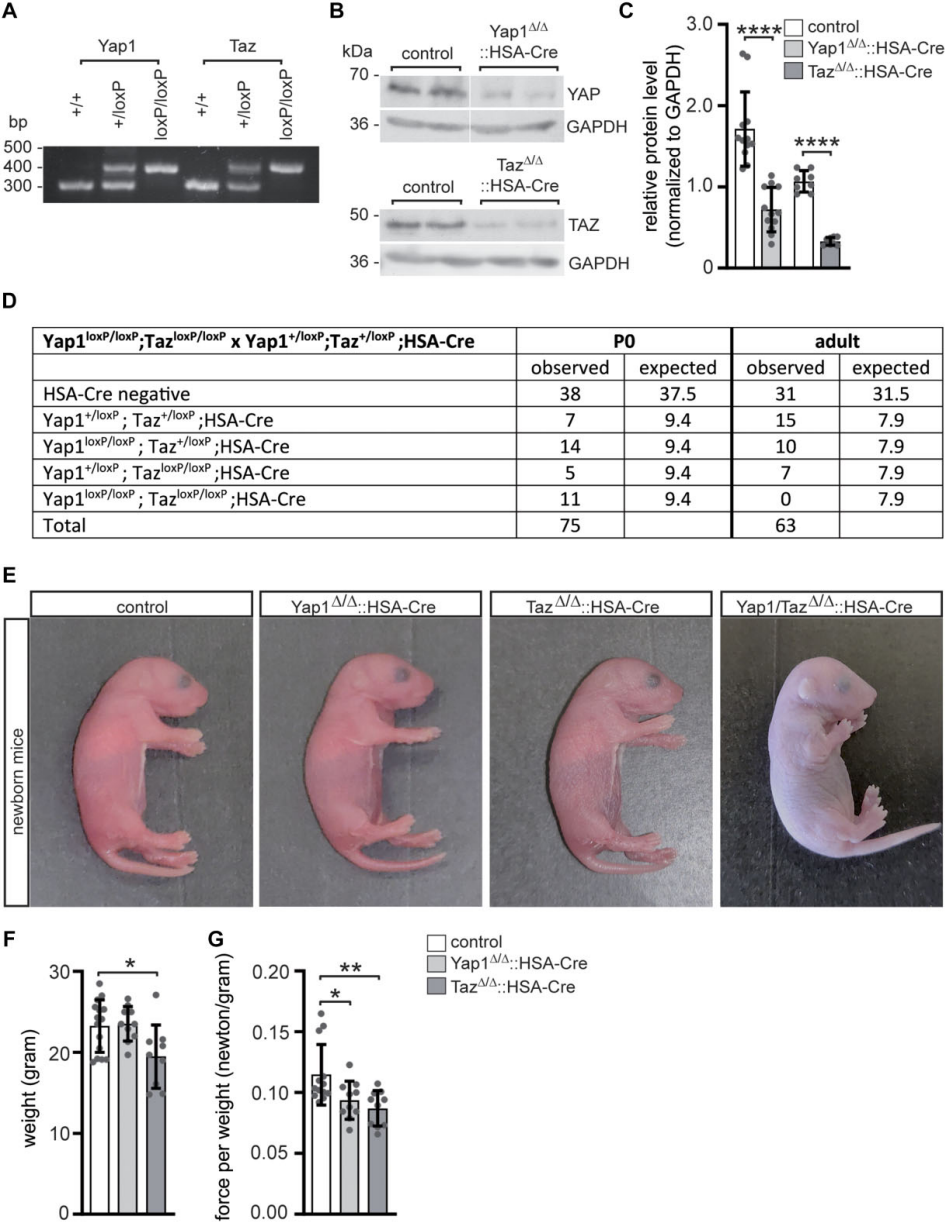
Decreased muscle grip strength was observed in conditional Yap1 or Taz knockout mice, while double knockout mice do not survive beyond birth. (**A**) The genotyping results of heterozygous and homozygous *Yap1* or *Taz* floxed mice are presented, and those mice which were in addition HSA-Cre positive were identified as muscle-specific knockout mutants. (**B**) Western blot experiments were performed using muscle lysates from the gastrocnemius muscle, demonstrating a loss of YAP1 or TAZ protein in adult muscle-specific knockout mice. (**C**) Western blot images, depicted in (**B**), were used for quantifying protein levels, normalizing them to GAPDH, and representing them in a graph. (**D**) The table presents the anticipated and observed offspring numbers for various genotypes. It is worth mentioning that newborn double floxed *Yap1*/*Taz* mice with HSA-Cre do not survive. (**E**) Images of newborn mice show that double knockout mice appear blue colored and are cyanotic due to respiratory failure. (**F**) A graph is provided to display the weight of mice with distinct genotypes. (**G**) Muscle grip strength of mice was assessed and presented in relation to the total mouse weight. Note, both *Yap1* and *Taz* mutant mice exhibit lower grip strength on comparison with control mice. *N* ≥ 5 mice per genotype. Please refer to the color assignment of the columns in the diagrams (C) and (G).

In spite of many similarities between transcriptional co activators YAP1 and TAZ, existing evidence contradicts with the view of generally compensating each other's function. Constitutive *Yap1* knockout mice are embryonically lethal (65). Constitutive *Taz* knockout mice are viable but characterized by renal cysts, which lead to end stage kidney disease (66). To study the individual and concerted role of YAP1 and TAZ at NMJs in muscle cells, we generated knockout mice by breeding HSA::Cre reporter mice, which express Cre recombinase under the control of the human skeletal actin (HSA) promoter (67), with floxed *Yap1* and/or *Taz* mice (51). PCR-based genotyping analysis ascertained identification of heterozygous and homozygous floxed alleles (Figure 2A). Accordingly, YAP1 and TAZ protein amounts were significantly reduced in hind limb muscle lysates of muscle-specific mutant mice (Figure 2B, C). Surprisingly, not all offspring genotypes did follow mendelian distribution (Figure 2D). While individual single and double mutant mice were detectable at embryonic stage, double knockout mice were not viable after birth (Figure 2D, E). Immediately after birth, double knockout mice were of cyanotic appearance due to inability to breath (Figure 2E) and their stomachs were never observed being filled with milk. At adulthood, the body weight of individual single mutant mice was not changed in *Yap1* mutant mice compared to control mice, but slightly reduced in *Taz* mutant mice (Figure 2F). However, when muscle strength was measured using a newton meter assay, it turned out that force per weight was slightly but significantly reduced in *Yap1*, and also in *Taz*, knockout mice (Figure 2G). No difference was detected between wild type (*Yap1*^+/+^, *Taz*^+/+^) and heterozygous mice with one deleted *Yap1* or *Taz* allele (*Yap1*/*Taz*^+/Δ^, HSA-Cre) or mice with floxed *Yap1* or *Taz* alleles (*Yap1*/*Taz*^+/loxP^, *Yap1*/*Taz*^loxP/loxP^), arguing against any haploinsufficiency. In this manuscript, control mice (*Yap1*/*Taz*^+/loxP^, *Yap1*/*Taz*^loxP/loxP^ or *Yap1*/*Taz*^+/Δ^::HSA-Cre), were compared with corresponding muscles of homozygous skeletal muscle conditional knockout *Yap1*/*Taz* mice (*Yap1*/*Taz*^Δ/Δ^::HSA-Cre) of the same litter. These findings reveal diverse muscle-specific phenotypes in individual *Yap1* or *Taz* mutant mice. The double knockouts are probably unable to breath and die shortly after birth.

### Regulation of muscle-specific gene transcription by electrical activity in adult individual muscle-specific Yap1 or Taz knockout mice

**Figure 3.**
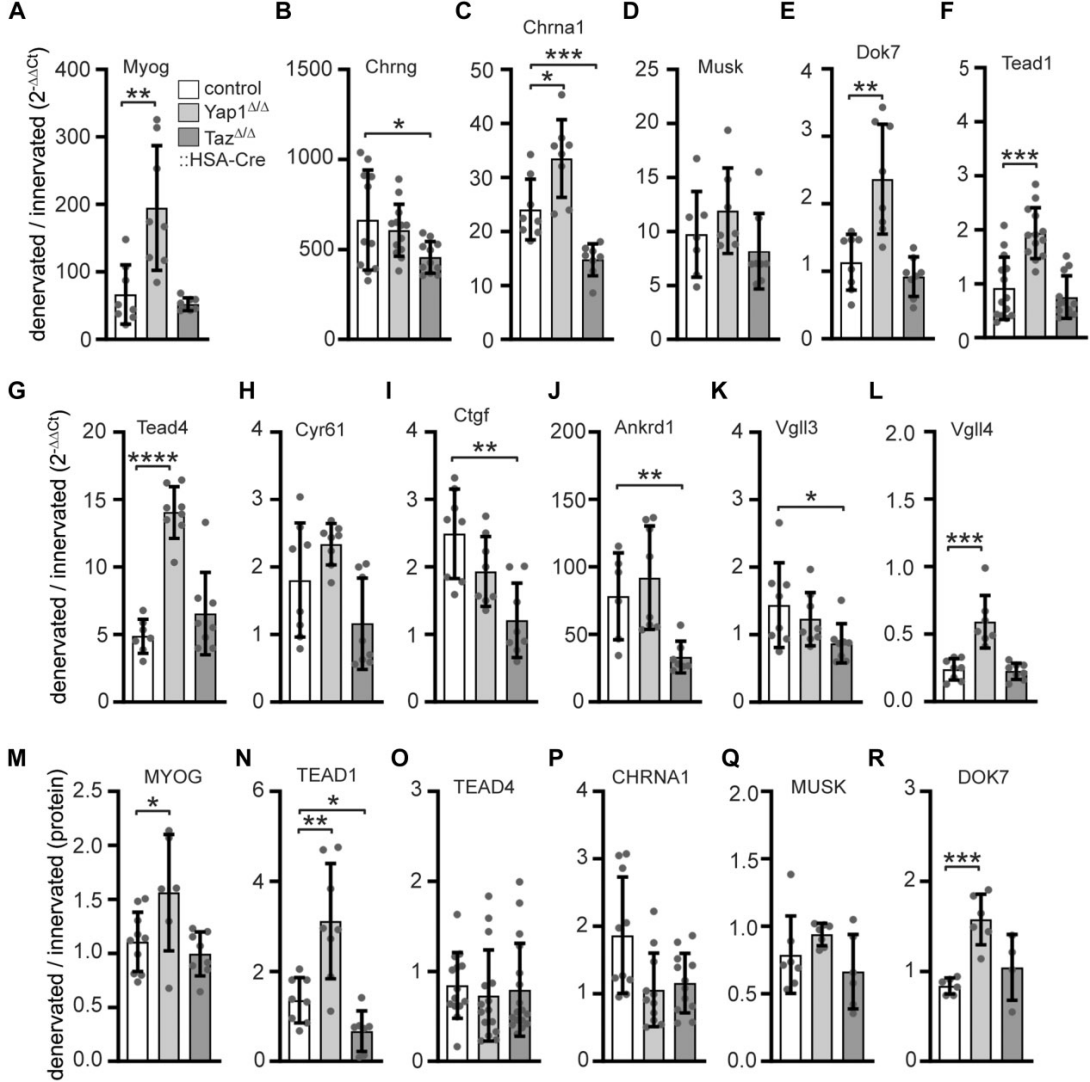
Comparison of transcript levels of myogenic genes upon denervation between adult control, Yap1, or Taz knockout extensor digitorum longus muscles. Transcript levels of myogenic genes were evaluated using qPCR on RNA samples from the extensor digitorum longus muscles of mice that were denervated for 5 days. After normalization values were calculated as the ratio of denervated to innervated for each genotype. The following myogenic genes were explored, (**A**) *Myog*, (**B**) *Chrng*, (**C**) *Chrna1*, (**D**) *Musk*, (**E**) *Dok7*, (**F**) *Tead1*, (**G**) *Tead4*, (**H**) *Cyr61*, (**I**) *Ctgf*, (**J**) *Ankrd1*, (**K**) *Vgll3*, (**L**) *Vgll4*. Several genes are significantly upregulated in *Yap1* knockout muscle when compared to control; striking *Dok7* is upregulated upon denervation in *Yap1* knockout mice, but this is known not to be the case in wild-type mice. Transcript level changes were confirmed at their protein level by quantifying several markers, like (**M**) MYOG, (**N**) TEAD1, (**O**) TEAD4, (**P**) CHRNA1, (**Q**) MUSK, (**R**) DOK7. Experiments were carried out on *N* ≥ 3 mice, and each qPCR was performed ≥ three times in duplicate. Please refer to the color assignment of the columns in the diagram (A).

Postsynaptic gene regulation in skeletal muscle fibers requires the action of myogenic transcription factors MYOD1 (MyoD) and MYOG (Myogenin). MYOG induces postsynaptic transcription in the absence of innervation or lack of electrical activity due to denervation (68). To analyze whether absence of YAP1 or TAZ impacts *Myog* regulation by electrical activity, hind limb muscles of control and *Yap1* or *Taz* knockout mice were denervated for 5 days and myogenic gene expression studied in comparison with innervated contralateral hind limb muscles by qPCR. *Myog* transcript levels were upregulated by > 66-fold in control, with no noteworthy distinction observed between control and *Taz* knockout hind limb muscles. Conversely, an upregulation of > 194-fold in *Myog* transcript levels was detected in *Yap1* knockout muscles (Figure 3A). The common denervation marker *Chrng* was significantly up-regulated by >662-fold in hind limb muscles of control mice (Figure 3B). A similar increase was observed in *Yap1* knockout muscles, but *Chrng* denervation response was significantly lower in *Taz* knockout muscles (Figure 3B). Next, transcript amounts of common postsynaptic genes, like *Chrna1*, *Musk*, and *Dok7* were analyzed (Figure 3C–E). *Chrna1* was strongly upregulated in denervated hind limb muscles of control mice (Figure 3C); even more in *Yap1*, but significantly less in *Taz* knockout muscles (Figure 3C). *Musk* was upregulated in control mice like shown before (69), and again a little higher in *Yap1* and lower in *Taz* knockout muscles, although these changes were not statistically significant (Figure 3D). As previously reported, *Dok7* was not upregulated in hind limb muscle of control mice (Figure 3E) (70). *Dok7* was also not upregulated in *Taz* knockout muscles, but significantly upregulated in *Yap1* knockout muscles (Figure 3E). Next, we investigated *Tead* transcript levels in denervated hind limb muscles. *Tead2* and *Tead3* were not upregulated and transcript amounts quite low, like in control mice (Figure 1E, F; data not shown). *Tead1* was only upregulated in *Yap1* and not in *Taz* knockout muscles (Figure 3F), while *Tead4* was strongly upregulated in control, *Yap1* and *Taz* knockout muscles (Figure 3G). *Tead4* transcript levels were > 3-fold higher upregulated in *Yap1* knockout in comparison with control and *Taz* knockout muscles (Figure 3G). Investigations of transcript levels of typical TEAD target genes revealed that *Cyr61* and *Ctgf* are upregulated upon denervation in control and *Yap1*, but not in *Taz* knockout muscles (Figure 3H, I). *Ankrd1* was upregulated in muscles of all genotypes by more than > 78-fold, but significantly less upregulated in *Taz* knockout muscles (>33-fold) (Figure 3J). We asked whether after denervation transcript levels of the TEAD repressors *Vgll3* and *Vgll4* might be affected in the absence of *Yap1* or *Taz*. *Vgll3* was not much changed in the absence of *Yap1*, but significantly downregulated in *Taz* knockout muscles (Figure 3K), while *Vgll4* was downregulated in all muscles, but significantly less in *Yap1* knockout muscle (Figure 3L). Additionally, transcript level changes were confirmed by their corresponding protein amounts using lysates of innervated and denervated muscle tibialis anterior of both control and knockout mice (Figure 3M–R). Overall, the upregulation of transcript levels of several but not all analyzed genes in *Yap1* knockout muscles compared to control and *Taz* knockout muscles suggests that *Yap1* and *Taz* do not phenocopy each other.

### Structural and functional NMJ deficits of adult muscle-specific Yap1 or Taz knockout mice

**Figure 4.**
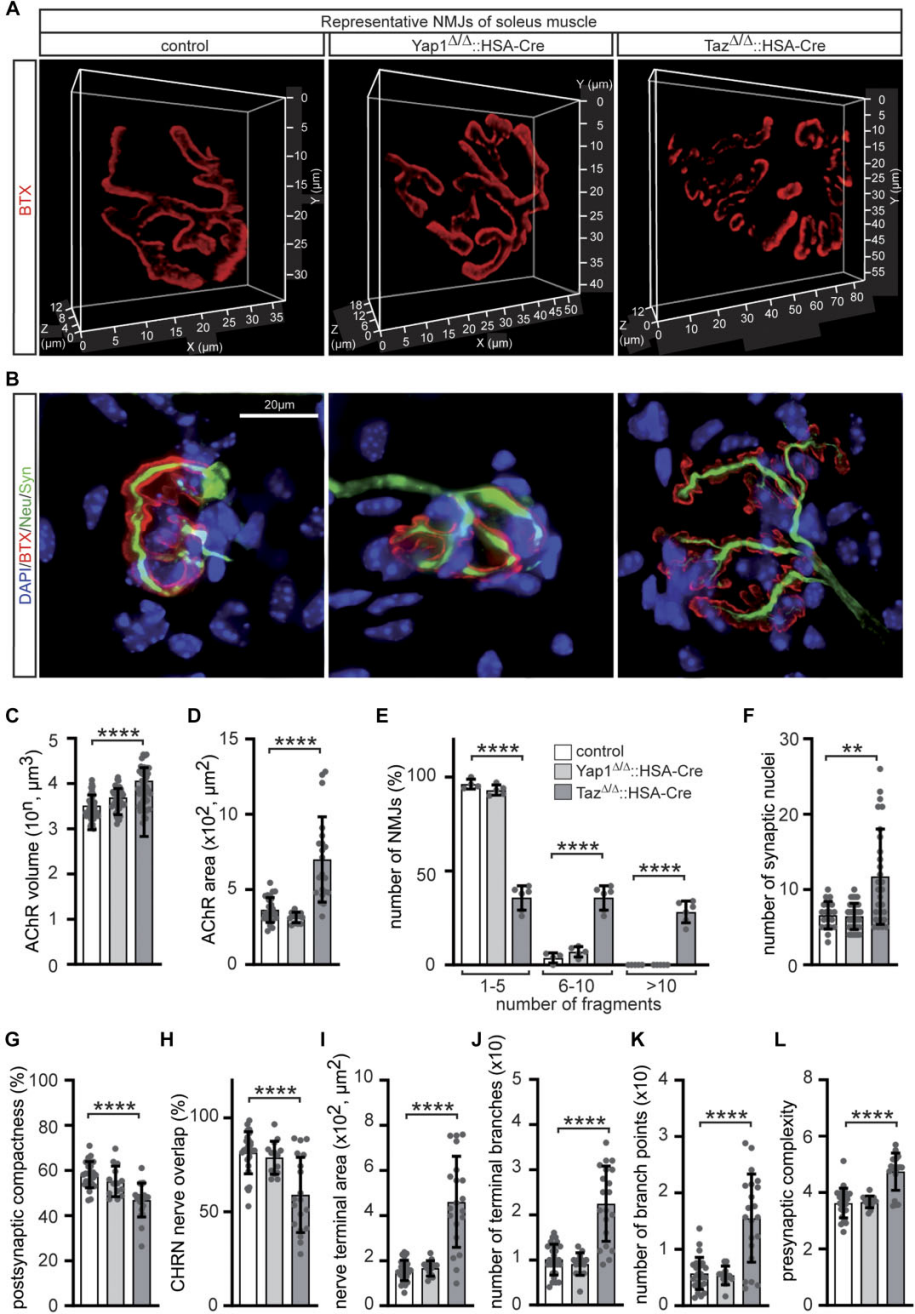
NMJs of Yap1 or Taz knockout mice exhibit fragmentation, increased size, a higher number of synaptic nuclei, and reduced overlap with nerve endings. NMJs of the soleus muscles of mice were stained with BTX, DAPI, and nerve markers, followed by 2D and 3D imaging. (**A**) Representative 3D images of control, *Yap1*, and *Taz* knockout mice are depicted. (**B**) High-resolution 2D images of representative NMJs were labeled by BTX, Neurofilament (Neu), and Synaptophysin (Syn). It is worth noting that NMJs in *Taz* knockout muscles appear fragmented, and more synaptic nuclei accumulate underneath the BTX-stained NMJs. (**C, D**) The volume and surface area of NMJs were quantified with 3D images for control, *Yap1* or *Taz* knockout mice and presented as graphs. Note, NMJs of *Taz* knockout mice were larger in volume and surface area. (**E**) Fragmentation degree of *Yap1* or *Taz* mutant mice in comparison with control is summarized in a graph. Note, indicative NMJ fragmentation was analyzed in *Yap1* knockout mice, while *Taz* knockout mice showed a significant increase of fragmentation. (**F**) The number of synaptic nuclei is significantly elevated in *Taz* mutant mice in comparison to control mice. (G-L) Additional morphological parameters of NMJs were characterized using aNMJ-morph algorithm, comparing control with *Yap1* or *Taz* knockout muscles: following parameters were analyzed, (**G**) postsynaptic compactness, (**H**) CHRN cluster-nerve overlap, (**I**) nerve terminal area, (**J**) number of terminal branches, (**K**) number of branch points, (**L**) presynaptic complexity. Note that all nerve parameters of *Taz* knockout NMJs exhibit a significant increase when compared with controls. *N* ≥ 50 NMJs per soleus per mouse per genotype were analyzed. Note, the color assignment information of the columns is represented by panel (E).

To investigate the underlying mechanisms of impaired grip strength of muscle-specific *Yap1* or *Taz* mutant mice, we further characterized the morphology of their NMJs. In aging or disease, physiologically pretzel-shaped mature NMJs have been described to be fragmented (71). By employing quantitative 3D imaging a significant increase of the BTX labeled volume and surface area of NMJs was detected in *Taz* mutant mice (Figure 4A, C, D). As previously reported (33), NMJ fragmentation was not detected in *Yap1* knockout mutant mice (Figure 4A, B, E). However, the NMJs of *Taz* mutant mice were heavily fragmented (Figure 4A, B, E). We also analyzed the number of synaptic nuclei in mutant mice (Figure 4F) which are transcriptionally specialized mainly expressing genes encoding for synaptic components (72). The number of synaptic nuclei was not changed in *Yap1* mutant mice, but significantly increased in *Taz* mutant mice (Figure 4F). The identity of additional synaptic myonuclei in *Taz* mutant mice was confirmed demonstrating their subcellular localization in muscle fibers spatially underneath by 3D imaging (suppl. Figure 1). However, it was not examined whether these additional subsynaptic nuclei in *Taz* knockout muscles transcribe *Chrn* genes. We asked whether motor nerve ending overlap with NMJs is impaired in *Yap1* or *Taz* knockout muscles in comparison with controls. We used a Fiji-based macro for analysis of NMJ morphology, called aNMJ-morph (46). While postsynaptic compactness and CHRN nerve overlap was significantly reduced in *Taz* knockout mice (Figure 4G, H), nerve terminal area, number of terminal branches and branch points, and presynaptic complexity were indicatively upregulated in *Taz* knockout muscles (Figure 4I-L).

**Figure 5.**
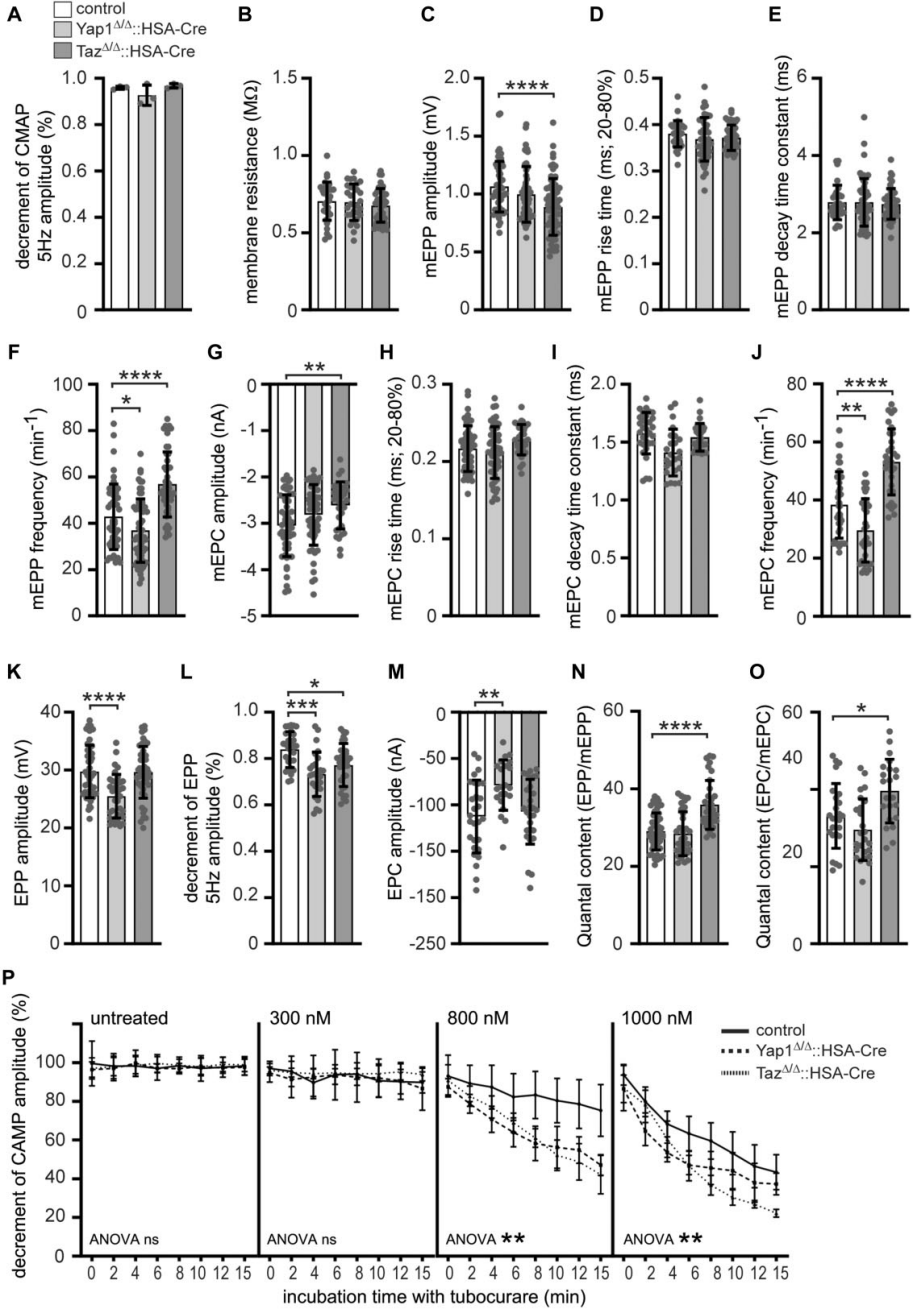
Characterization of neural transmission of Yap1 or Taz knockout diaphragm. Electrophysiological recordings were conducted on diaphragms of adult mice. Graphs present the analysis of the various parameters: (**A**) decrement of compound muscle action potential amplitude at 5 Hz, (**B**) membrane resistance, (**C**) miniature endplate potential amplitude, (**D**) miniature endplate potential rise time, (**E**) miniature endplate potential decay time constant, (**F**) miniature endplate potential frequency, (**G**) miniature endplate current amplitude, (**H**) miniature endplate current rise time, (**I**) miniature endplate current decay time constant, (**J**) miniature endplate current frequency, (**K**) endplate potential amplitude, (**L**) decrement of endplate potential amplitude at 5 Hz, (**M**) endplate current amplitude, (**N**) Quantal content (EPP/mEPP), (**O**) Quantal content (EPC/mEPC). (**P**) CMAP was recorded using diaphragms under untreated conditions and in the presence of increasing concentrations (300, 800 and 1.000 nM) of d-tubocurarine. Knockout diaphragms showed a significantly higher decrement of CMAP, which was displayed at 800 nM d-tubocurarine, thus unmasking a reduced safety factor in the absence of *Yap1* or *Taz*. The analysis comprised of N ≥ 5 mice per genotype with >20 individual NMJs per muscle. ANOVA statistics were employed for d-tubocurarine experiments (**P**). Panel (A) presents information on the color assignment of the columns (**A**).

Next, we recorded diaphragm muscles of control and mutant mice to analyze whether the physiology of neuromuscular transmission at NMJs is impaired (Figure 5). Previously, *Yap1* mutant mice were reported to be characterized by neuromuscular transmission deficits (33). We recorded CMAPs, compound muscle action potentials that are triggered by consecutive nerve stimuli at 5 Hz, and did not observe any significant change of the decrement of amplitudes in mutant diaphragm muscles (Figure 5A). The membrane resistance values were comparable between different genotypes arguing for non-affected membrane integrity in mutant muscles (Figure 5B). However, recording of miniature endplate potentials (mEPP) and currents (mEPC) revealed a significant change of their frequencies (Figure 5F, J). While in *Yap1* mutant mice the mEPP and mEPC frequencies decreased, in *Taz* mutant mice a significant increase was detected (Figure 5F, J). Moreover, mEPP and mEPC amplitudes were different in *Taz* mutant mice arguing for impaired local depolarizations around endplates in response to spontaneous acetylcholine release (Figure 5C, G). Of note, mEPP and mEPC rise time and decay time constants were not changed in either mutant in comparison to control (Figure 5D, E, H, I). On the other hand, EPP and EPC amplitudes, local responses at NMJs to nerve stimulation, were decreased in *Yap1* mutant mice (Figure 5K, M). Run down experiments demonstrated a decrease of EPP decrement for both *Yap1* and *Taz* mutant mice (Figure 5L). In agreement, the quantal content, the number of acetylcholine quanta released upon a single nerve impulse, was increased for *Taz* mutant mice (Figure 5N, O).

To provide further evidence for impaired neuromuscular transmission we evaluated the safety factor, which reflects the fact that the threshold required to generate a muscle action potential was exceeded by the excitatory effect generated by nerve stimulation (73). We carried out compound muscle action potential (CMAP) measurements on control and mutant diaphragms in the presence of increasing concentrations of d-tubocurarine in order to monitor the effect of a partial block of CHRNs like described before (44). Treatment with d-tubocurarine led to a dose-dependent strong decrease of the decrement in response to repetitive stimuli in *Yap1* and *Taz* knockout but not in control muscles (Figure 5P). Obviously, a concentration range of 800 nM d-tubocurarine resulted in a strong decrease of the decrement in mutant muscles, while higher concentrations of d-tubocurarine (≥1000 nM) were presumably blocking too many CHRNs, making it impossible to detect a change between controls and mutants (Figure 5P). Altogether, our data demonstrate structural and functional impairments at the NMJs of adult *Yap1* or *Taz* mutant mice.

### In neonatal Yap1/Taz double knockout mice, the endplate bands are significantly disorganized, accompanied by a lower transcription level of synaptic genes

**Figure 6.**
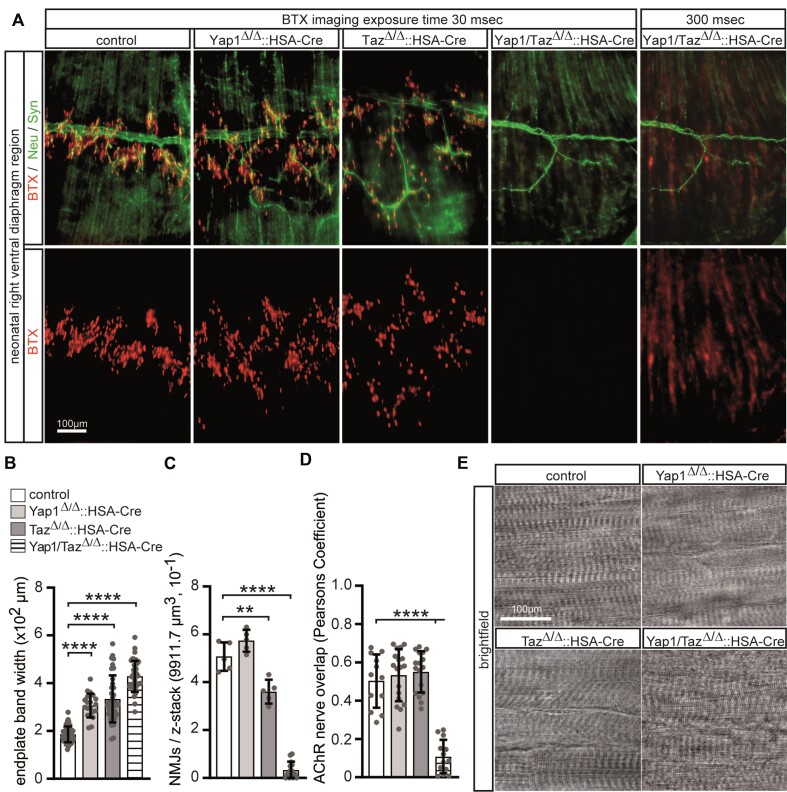
Neonatal diaphragm endplate bands of Yap1 or Taz or double knockout mice exhibit enlargement, moreover, CHRN clusters are almost not present in double knockout mice and barely overlapped with nerve endings. Diaphragms of newborns were dissected and BTX, Neurofilament (Neu) and Synaptophysin (Syn) stained. (**A**) Immunofluorescence microscopy images of typical stainings of the right ventral diaphragm regions of control and knockout mice are shown. The upper row presents a co-stain of BTX and Neu/Syn, while the lower row shows only the BTX stain to better visualize endplate band width enlargements. Notably, BTX stained NMJs of double knockout mice are significantly lower in number, irregularly distributed, and less bright in immunofluorescence microscopy. It required a much higher exposure time (∼10-fold) to make BTX staining visible. (**B**) The graph summarizes endplate band width of control and knockout diaphragms. (**C**) Graph presents number of NMJs per 3D field of view in control and knockout diaphragms. Note, per view field less NMJs have been observed in Taz knockout diaphragms; double knockout diaphragms contain almost no NMJs. (**D**) The graph summarizes the overlap of NMJs by the nerve in control and knockout diaphragms. (**E**) Representative bright field images of fibers showed no apparent impairment of endplate band pattern of fibers of knockout mice in comparison with the control in the diaphragm muscles. For each genotype, *N* ≥ 3 mice were analyzed, and >20 individual NMJs were analyzed per muscle. Note the information on the color assignment of the columns presented in panel (T).

While individual muscle-specific *Yap1* or *Taz* mutant mice were viable through adulthood, double knockout mice were dying immediately after birth due to inability to breath (Figure 2D, E). We explored whether compromised clustering of CHRNs is the cause or is involved in neonatal lethality. BTX-labeled diaphragms of individual *Yap1* or *Taz* mutant mice, or double knockout mice, were whole mount imaged and analyzed (Figure 6A, C). At first glance, CHRN clusters of individual neonatal *Yap1* or *Taz* mutant mice looked plaque-shaped, as expected at this developmental stage, and of regular appearance in comparison with controls, but endplate bands were being of enlarged width (Figure 6A, B). With the same exposure times used to image CHRN clusters on diaphragms of control and single mutant mice, BTX-stained CHRN clusters were barely visible in double knockout diaphragms (Figure 6A). Using significantly higher exposure times to image double knockout diaphragms, a very low number of CHRN clusters with irregular morphology and localization, extending partly from one tendon of the fibers to the other tendon, were detected (Figure 6A, B). At higher magnification and additional nerve terminal staining, the plaque-shaped morphology of NMJs of double mutant diaphragms looked shattered (data not shown). The number of NMJs per Z-stack was not changed between *Yap1* mutant and control mice, but significantly decreased in *Taz* knockout mice and even more in the double knockout mice (Figure 6C). Moreover, the nerve terminals of individual *Yap1* or *Taz* mutant mice overlap with CHRN clusters like in controls (Figure 6A, D). The amount of nerve coverage was severely affected in double mutant mice in comparison to controls (Figure 6A, D). High-resolution bright field images of the diaphragms of control and mutants did not reveal any obvious disturbances regarding M and Z band patterning of diaphragm muscle fibers; if at all, irregularities were detected in double knockout muscles (Figure 6E). In summary, the impairments of NMJs in single mutant mice were significantly less severe in comparison to those in double mutant mice.

**Figure 7.**
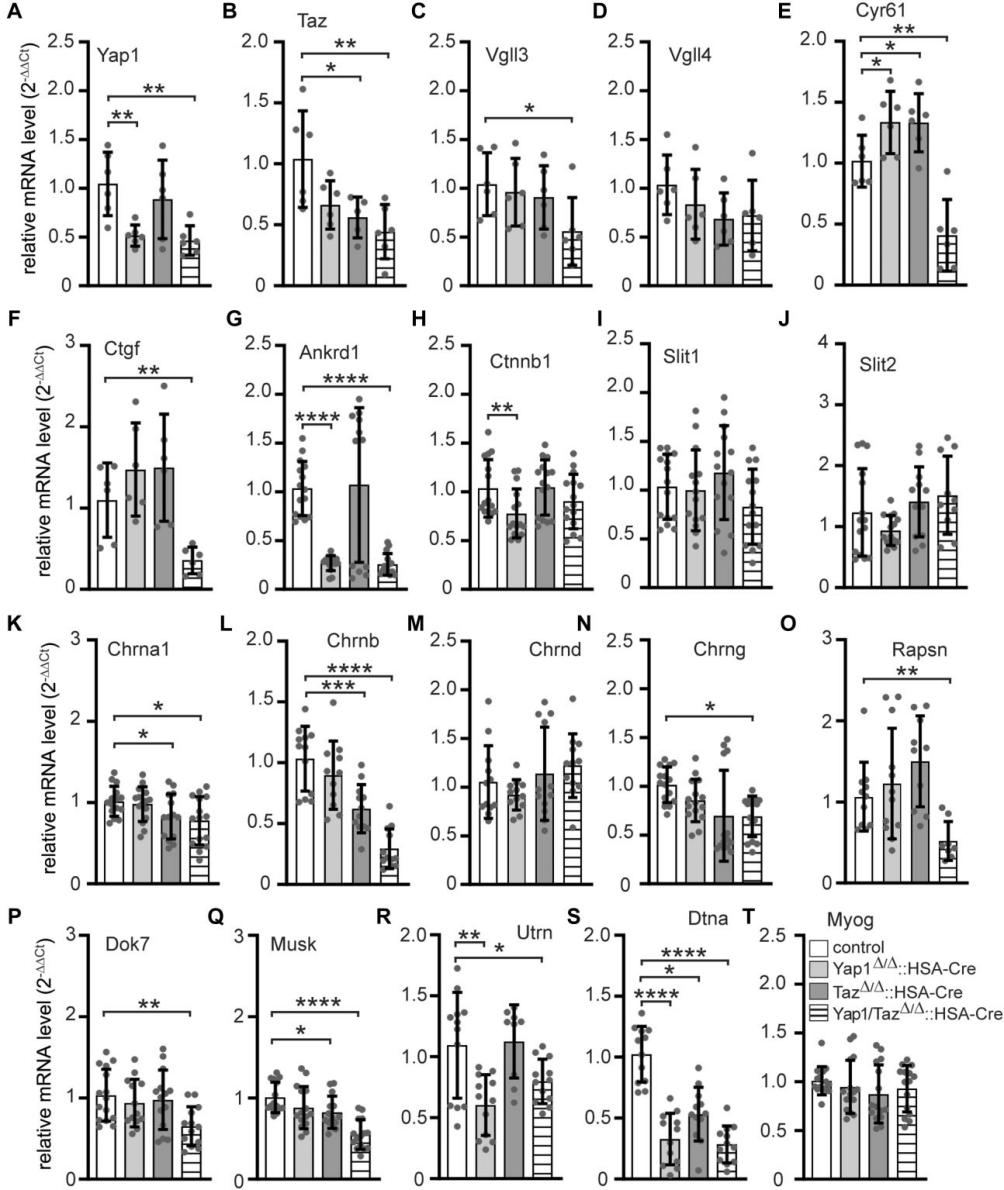
Comparison of transcriptome profiles of myogenic genes in hind limb muscles of control and knockout mice. Total RNA was extracted from the hind limb muscles of newborn mice. Transcript levels of various myogenic genes were analyzed using qPCR. (**A, B**) *Yap1* and *Taz* transcript levels were reduced in agreement with the genotype of the muscles. (**C, D**) Transcript levels of the TEAD repressors *Vgll3* and *Vgll4* were suggestively slightly reduced in the knockout muscles. (**E**–**G**) The transcript levels of TEAD target genes, *Cyr61*, *Ctgf* and *Ankrd1* were found to be downregulated in the double knockout muscles. (**H**–**J**) The transcript levels of the effectors of canonical Wnt signaling, including *Ctnnb1*, *Slit1* and *Slit2* were slightly changed in knockout muscles. (**K**–**S**) Transcript levels of synaptic genes such as *Chrna1*, *Chrnb*, *Chrnd*, *Chrng*, *Rapsn*, *Dok7*, *Musk*, *Utrn*, and *Dtna*, were mostly declined in double knockout muscles. (**T**) The transcript level of *Myog* did not show any alteration in knockout muscles. The experiments were analyzed using *N* ≥ 3 mice, and each qPCR was performed at least three times in duplicate. Note the information on the color assignment of the columns is as presented by panel (O).

We wondered whether lower fluorescence intensity of CHRN clusters might be related to lower transcriptional rates of synaptic genes in mutant mice. Hind limb skeletal muscles of newborn control and mutant mice were used to extract RNA for qPCR experiments. First, as expected we observed *Yap1* and *Taz* transcript amount lower in the respective mutant muscle tissues (Figure 7A, B). Second, the transcript amounts of TEAD repressors *Vgll3* and *Vgll4* were both, either indicatively or significantly, reduced in double knockout muscles (Figure 7C, D), though *Vgll4* transcript level was indicatively lowered the same strong in *Taz* knockout muscles (Figure 7D). Third, the transcript amounts of the typical YAP1/TAZ target genes *Ankrd1*, *Cyr61* and *Ctgf* were significantly low in the double mutant muscle tissues, but less reduced, not reduced, or even increased analyzing the RNA of single mutant muscles (Figure 7E–G). While transcript level of *Cyr61* was increased in *Yap1* and *Taz* knockout muscles (Figure 7E), *Ankrd1* was significantly decreased in *Yap1* knockout and double knockout muscles (Figure 7G). Fourth, we explored whether muscle YAP1 and TAZ regulate presynaptic differentiation, since these phenotypes resemble those in mice lacking *Ctnnb1* in the skeletal muscle (27,29,74). It was hypothesized that this similarity suggests *Ctnnb1* as a potential target of *Yap1* mutation (33) or might be indicative for a cross-talk between YAP1/TAZ-TEAD and canonical Wnt signaling (25). In fact, we analyzed *Ctnnb1* being transcribed at similar levels in *Taz* and double mutant neonatal mice in comparison with controls, less *Ctnnb1* was detected in *Yap1* mutant mice (Figure 7H). Fifth, muscle CTNNB1 is thought to regulate presynaptic differentiation by controlling expression of releasable factors, such as SLIT2 (29). We determined neither SLIT1, nor SLIT2, transcript levels being changed in all mutant neonatal muscles in comparison with controls (Figure 7I, J). Sixth, we detected significantly lower transcript levels of *Chrna1*, in *Taz* knockout muscles (Figure 7K), and reduced *Chrna1*, *Chrnb*, and *Chrng* transcript levels in double mutant skeletal muscles (Figure 7K, L, N) in comparison to controls. Seventh, since *Chrn* gene transcription and/or CHRN clustering require RAPSN, a postsynaptic cytoplasmic protein which participates in CHRN assembly, and active MUSK signaling, we looked for transcript amounts of *Rapsn*, *Musk*, and its adaptor *Dok7*; all were significantly lowered in double mutant muscle tissue (Figure 7O, P, Q). Eighth, the transcript of other typical postsynaptic genes was also reduced in double mutant muscle tissue, like *Utrn* and *Dtna* (Figure 7R, S). Ninth, the amount of late muscle fiber differentiation marker *Myog* was not reduced in muscles of mutant mice in comparison to control mice (Figure 7T). Altogether, our data support an involvement of YAP1/TAZ-TEAD dependent signaling to ensure proper transcription of synaptic genes.

### Ablation of Tead1 and Tead4 expression in myotubes impairs AGRN-dependent CHRN clustering

**Figure 8.**
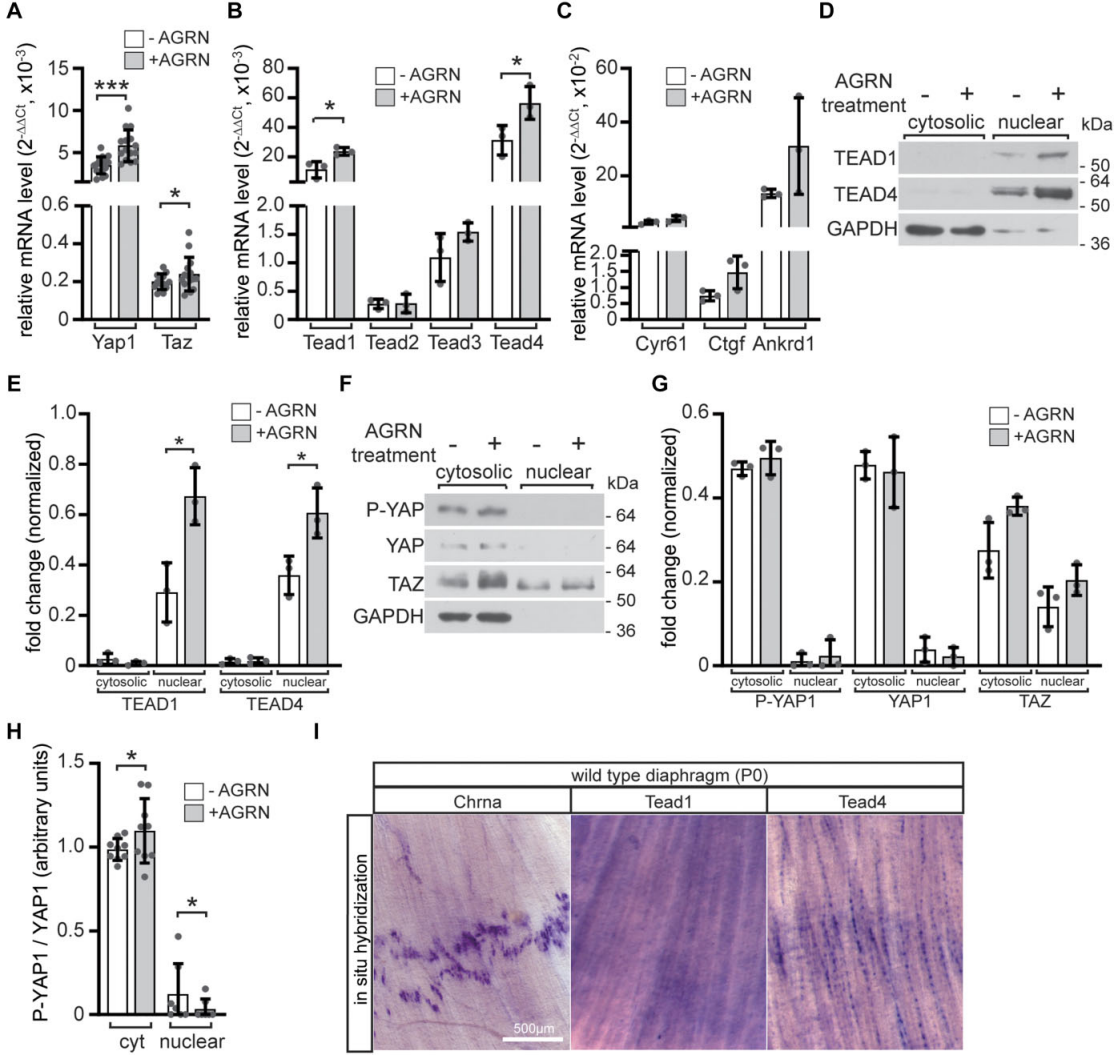
Neural derived AGRN stimulated the transcription of YAP1/TAZ-TEAD signaling members in cultured primary muscle and diaphragm muscles. Wild type primary muscle cells were differentiated into myotubes for 3 days. They were then treated with AGRN-conditioned media (42) for 16 h. After treatment, mRNA was extracted and quantified (A–C), or cells were lysed and cytosolic and nuclear fractions of the protein lysates were analyzed by SDS-PAGE and western blot (D–G). Myotube treatment with AGRN resulted in a significant increase in the expression of *Yap1* and *Taz* (**A**) as well as and *Tead1* and *Tead4* (**B**) in comparison with control cells, which were not exposed to AGRN. (**C**) The mRNA levels of TEAD target genes, *Cyr61*, *Ctgf* and *Ankrd1*, showed a tendency to increase in response to neural AGRN. (**D, F**) Representative Western blot images displayed cytosolic and nuclear fractions of protein lysates and the related graphs summarized the quantifications of these images (**E, G**). Note, TEAD1 and TEAD4 nuclear levels significantly increased after AGRN treatment compared to untreated control. There were no significant changes detected in the protein levels of YAP1 or Phospho-YAP1 (Ser 127). TAZ levels were slightly elevated after AGRN treatment, but not significantly. (**H**) The graph summarizes calculation of the ratio of P-YAP1 to YAP1 as shown in (F). (**I**) Images show *in situ* hybridization with riboprobes complementary to mRNAs of *Chrna1*, *Tead1* or *Tead4* on neonatal diaphragm muscles. Note, *Chrna1*, *Tead4* transcripts accumulate within the endplate zone at the center of the muscles. Each qPCR was performed in duplicate with a minimum of three set of cells.

To better understand which transcriptional mediators of the YAP1/TAZ-TEAD pathway are involved in the regulation of postsynaptic gene expression, we analyzed whether expression of any of these mediators directly responds to activation of the AGRN/LRP4/MUSK signaling. Cultured wild type primary muscle cells were differentiated to myotubes and AGRN/LRP4/MUSK signaling was stimulated by addition of conditioned media containing neural AGRN. AGRN treatment significantly elevated the transcript levels of *Yap1* and *Taz* (Figure 8A), as well as *Tead1* and *Tead4* (Figure 8B). The expression of the typical TEAD target genes *Cyr61*, *Ctgf* and *Ankrd1* expression was elevated, but not significantly changed (Figure 8C). The transcriptional changes were confirmed by significant AGRN-induced increase in protein levels of TEAD1 and TEAD4 (Figure 8D, E) by Western blot of the nuclear fraction of protein lysates of AGRN treated wild type primary myotubes, while assessment of nuclear levels of the other markers YAP1, phospho-YAP1, or TAZ showed no significant changes (Figure 8F, G). Changed levels of active YAP1 upon AGRN stimulation in muscle cells were confirmed by calculation of P-YAP1/YAP1 amounts (Figure 8H). We performed *in situ* hybridization with riboprobes targeting transcripts of *Tead1* and *Tead4* using diaphragm muscles of newborn wild type mouse pups to investigate their localization in skeletal muscle *in vivo*. *Chrna1* expression delineates the endplate zone containing the NMJs (Figure 8I). While *Tead1* transcripts were equally distributed along the muscle fiber length, *Tead4* transcripts were prominently concentrated at the endplate zone (Figure 8I).

**Figure 9.**
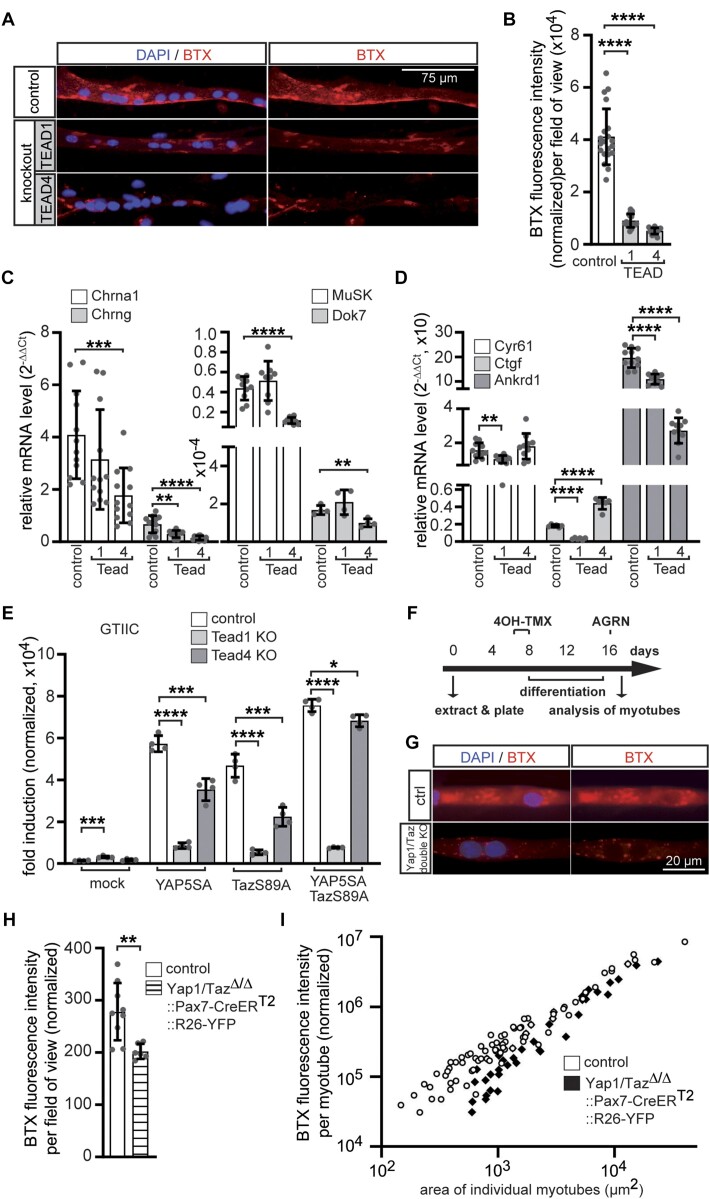
In Tead1, Tead4, and Yap1/Taz knockout primary myotubes, AGRN-induced CHRN clustering and reduced synaptic gene expression. (**A**) Control and CRISPR knockout cells were differentiated to myotubes for 5 days and treated with AGRN-conditioned media for 16 h before PFA fixation and staining with BTX and DAPI. (**B**) CHRN clusters on myotubes were quantified with Fiji image processing from 20-fold objective images and normalized to number of nuclei in the myotubes. *Tead1* and *Tead4* knockouts showed a severe reduction of CHRN clusters, if compared to control, as observed by reduction of BTX fluorescence intensity. *N* ≥ 2 clones per knockout, *N* ≥ 3 sets of cells and *N* ≥ 16 images per set and sample. (**C**) Control and CRISPR knockout primary muscle cells were differentiated to myotubes for 5 days, RNA was extracted and transcript levels of *Chrna* and *Chrng*, *Musk* and *Dok7* were assessed by qPCR. *Tead4* CRISPR knockout cells exhibited the strongest effect on expression of these genes in comparison to control. *N* ≥ 3 sets of cells, qPCR were performed ≥ three times in duplicate for each set of cells. (**D**) Using the same cells like in (C) the profile of common TEAD target genes *Ankrd1*, *Cyr61* and *Ctgf* was investigated. Note, transcription of TEAD target genes showed a strange pattern after loss of either *Tead1* or *Tead4*, compared to control. While transcript levels of all three TEAD targets decreased in *Tead1* knockout cells, in *Tead4* knockout cells *Cyr61* was unchanged, *Ctgf* increased and *Ankrd1* decreased. (**E**) To understand more about TEAD mediated target gene expression in CRISPR *Tead1* and *Tead4* knockout cells, the GTIIC luciferase reporter was transfected together with expression plasmids encoding constitutive active mutants of YAP1 (YAP5SA), TAZ (TAZS89A), or both, into control and CRISPR knockout cells. Note, the ability of the transcriptional co-activators to stimulate the GTIIC reporter was significantly impaired in the *Tead1* knockouts and moderately impaired in the *Tead4* knockouts after transfecting either one of the two constitutive active co-activators. However, co-transfection of constitutive active mutants of YAP1 and TAZ together into *Tead4* knockout cells almost fully rescued luciferase activity levels to control levels. (**F**) To confirm that transcriptional co-activators YAP1 and TAZ mediate TEAD dependent transcription of synaptic genes, primary muscle cultures were established for *Yap1* and *Taz* double knockout muscle cells by extracting the satellite cells from Yap1/Taz^loxP/loxP^::Pax7-CreER^T2^::R26R^YFP/+^ mice and incubating the cells with 4-hydroxy tamoxifen (4OH-TMX). The diagram shows the schedule of 4OH-TMX treatment and incubation with neural AGRN in satellite cells isolated from control or knockout mice. (**G, H**) After differentiation of the myoblasts to myotubes on laminin coated plates, CHRN cluster formation was detected by BTX staining and quantified using Fiji. Note, BTX fluorescence intensity per field of view was strongly reduced in *Yap1* and *Taz* double knockout myotubes. Exposure time to take images was 10-fold higher in double knockout cells compared with controls arguing for very low amount of CHRN clusters. (**I**) BTX fluorescence intensity as plotted against the area of individual myotubes. *N* ≥ 3 set of cells, N ≥ 16 images per set and sample.

To study the neuromuscular phenotype of canonical Wnt and YAP1/TAZ-TEAD signaling effectors, which expression is elevated in primary myotubes in response to AGRN, we generated several independent bi-allelic primary knockout muscle cells for *Tead1* or *Tead4* genes using CRISPR/Cas9 mediated gene editing (suppl. Figure 2). Several clones of *Tead1* and *Tead4* CRISPR knockout cells were used to investigate their ability to form CHRN clusters in response to neural AGRN. Equal numbers of respective cells were plated, differentiated for 5 days and subsequently incubated with AGRN-conditioned media for 16h to induce clustering of CHRNs. The CHRN clusters were visualized with BTX and cell nuclei with DAPI (Figure 9A). *Tead1* and *Tead4* CRISPR knockout myotubes showed a strong impairment of AGRN-induced CHRN clustering, as proven by significant reduction in normalized total fluorescence intensity (Figure 9A, B). Aiming to understand how the loss of *Tead1* or *Tead4* impairs CHRN clustering, we quantified the expression of genes encoding the *Chrna1* and *Chrng*, *Musk*, and *Dok7* in differentiated *Tead1* and *Tead4* CRISPR knockout primary muscle cells (Figure 9C). While *Tead1* knockout only affected expression of *Chrng*, *Tead4* knockout significantly reduced the expression of all four synaptic genes (Figure 9C). Overall, the strongest reduction in synaptic gene expression compared to control was observed in *Tead4* knockout muscle cells, which was also reflected in the inhibition of their AGRN-induced CHRN clustering ability (Figure 9A, B). This indicates that the reduced amount of AGRN-induced CHRN clustering in the CRISPR knockout cells (Figure 9A, B) is at least partly a consequence of reduced expression of the involved synaptic genes (Figure 9C). To gain more insights into how transcription of TEAD target genes was affected in the absence of *Tead1* or *Tead4*, we analyzed the transcript amounts of the common TEAD target genes *Ankrd1*, *Cyr61* and *Ctgf* in the respective CRISPR knockout cells. Transcription of TEAD target genes *Ankrd1*, *Cyr61* and *Ctgf* was reduced in *Tead1* knockout muscle cells (Figure 9D), while *Tead4* knockouts differentially affected the expression of TEAD target genes (Figure 9D). Moreover, we investigated the ability of *Tead1* and *Tead4* CRISPR knockout cells to stimulate a TEAD-dependent luciferase reporter (GTIIC). After transfection of the reporter into wild type myoblasts and differentiation to myotubes, together with either constitutive active YAP5SA, TAZS89A, or both, significantly induced reporter activity was detected (Figure 9E). The ability of *Tead1* and *Tead4* CRISPR knockout myotubes to stimulate the luciferase reporter upon transfection of constitutive active YAP1/TAZ mutants was significantly diminished (Figure 9E), however the effect was much more pronounced in *Tead1* knockouts than *Tead4* knockouts (Figure 9E). The co-transfection of both, constitutive active YAP5SA and TAZS89A into *Tead4* CRISPR knockout cells almost completely compensated luciferase values to control levels (Figure 9E). We asked whether knockout of the transcriptional co-activators *Yap1* and *Taz* would also affect regular CHRN clustering in cultured cells, like observed for *Tead1* and *Tead4* knockout cells (Figure 9A, B). Satellite cells were extracted from double knockout mutant mice additionally bearing a Pax7 promoter controlled Cre recombinase-ER^T2^. After 4-hydroxy tamoxifen (4OH TMX)-dependent knockout of the *Yap1* and *Taz* genes in cultured primary myoblast, the cells were fused to myotubes and incubated on plates coated with laminin to stimulate AGRN independent CHRN clustering (Figure 9F), like described before (75). A significant reduction of the amount of CHRN clusters stained by BTX was detected in the double mutant myotubes in comparison to controls (Figure 9G–I).

### TEAD binding sites regulate expression of Chrna1, Musk and Dok7

**Figure 10.**
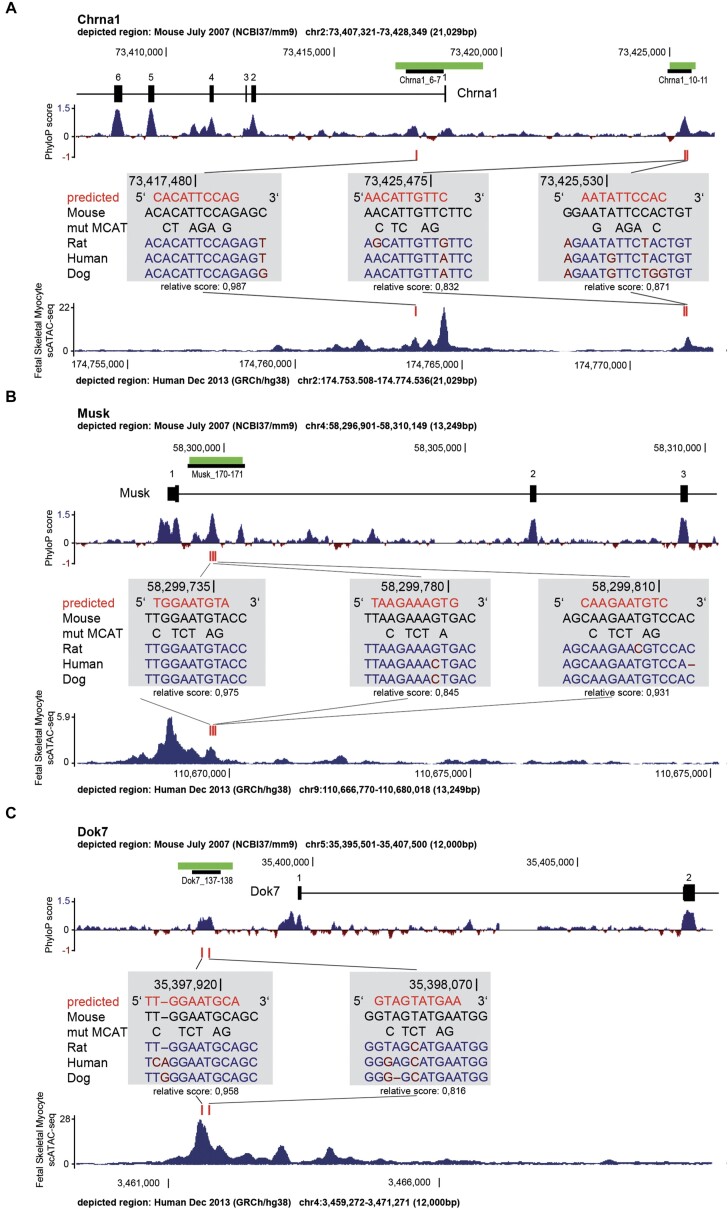
TEADs bind to M-CAT motifs in evolutionarily conserved and open chromatin regions of postsynaptic key genes, such as Chrna1, Musk and Dok7. A search for putative M-CAT motifs for TEAD transcription factors was performed in evolutionary conserved regions of synaptic genes previously occupied by TEAD4 in ChIP-Seq experiments in differentiated C2C12 muscle cells (47). Several putative M-CAT binding sites that were highly conserved among mammalian species were identified in genes such as *Chrna1* (**A**), *Musk* (**B**) and *Dok7* (**C**) along with others (listed in Supplementary Table S3). The genomic loci are displayed indicating highly conserved areas, such as gene exons or possible regulatory regions, with PhyloP conserved score peaks. TEAD4-occupied sites are presented as bright green rectangles, while putative TEAD4 binding sites are labeled in red and presented in boxes with multiple alignments of respective genomic sequences from various mammalian species. The search for TEAD4 putative binding sites was performed with the JASPAR 2018 Scan function (TEAD4 matrix profile ID: MA0809.1) and the default relative score profile threshold of 80%. The relative scores of each site are specified under the multiple alignment boxes. Further, scATAC-seq dataset was explored to examine open chromatin regions of above-mentioned genes. Notably, the identified M-CAT motifs are located in open chromatin regions of human skeletal myocytes.

**Figure 11.**
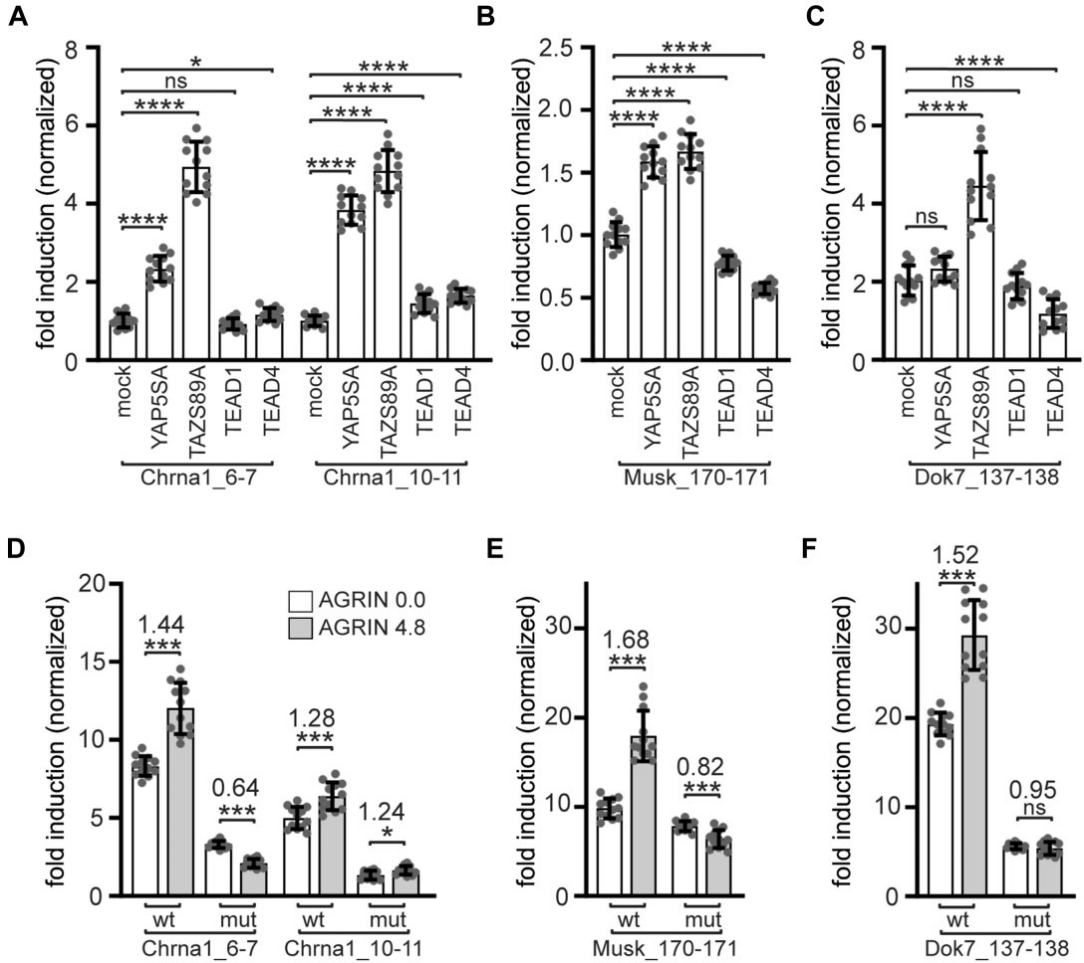
M-CAT motifs have been identified in postsynaptic key genes, such as Chrna1, Musk and Dok7 to enhance their transcription. The genomic regions labeled black rectangles in Figure 10A-C were cloned into the pGL4.20-Hsp68min vector located upstream of the luciferase ORF, to investigate whether TEADs could activate the expression of the luciferase reporter. Two different approaches were employed for the transfection of luciferase reporter constructs. They were co-transfected with one expression plasmid each (Tead1, Tead4, constitutively active Yap5SA, or TazS89A) into cultured C2C12 muscle cells. Luciferase activity was then assessed in cell lysates of proliferating cells 48h after transfection (**A–C**). Alternatively, C2C12 cells were transfected with one reporter each, differentiated for 5 days, and incubated with inactive or active neural AGRN for 16 h before lysing the cells and performing luciferase assays (**D–F**). The constitutive active TAZS89A alone showed the strongest stimulation in all reporters. All M-CAT motifs were mutagenized as shown in Figure 10A–C. The reporters with mutagenized M-CAT motifs were stimulating luciferase reporters significantly less compared to non-mutagenized reporters (D–F). Important, while non-mutagenized M-CAT motif regulated reporters were induced by neural AGRN, whereas mutagenized M-CAT motifs abolished the stimulation of reporters by neural AGRN. *N* ≥ 3 sets of cells were subjected to a luciferase assay, with each assay performed ≥ three times as triplicate. Note the information on the color of the columns is assigned as shown by panel (D).

TEAD transcription factors mediate transcriptional activity by binding to so-called M-CAT motifs of DNA, which are also present in many muscle genes (76) and have also been found in regulatory regions of synaptic genes (77,78). A recent study investigating the myogenic role of TEAD transcription factors provided ChIP-Seq data on the genomic occupancy by TEAD1 and TEAD4 in undifferentiated and differentiated C2C12 muscle cells (47). We examined those data for genomic occupancy by TEAD1 and TEAD4 in promoters or enhancers of synaptic genes. We found TEAD4-occupied regions close to the genomic loci belonging to genes, like *Chrna1*, *Chrng*, *Musk*, *Utrn* and *Dok7* in dataset of differentiated C2C12 cells, but not in non-differentiated C2C12 cells (Supplementary Table S2). We focused our attention on *Chrna1*, *Musk*, and *Dok7* as they represent three typical key players at the adult postsynaptic apparatus. According to the published ChIP-Seq datasets genomic loci of these three players were occupied only by TEAD4, but not by TEAD1, despite very similar DNA binding motifs among TEAD transcription factors (47). Using the JASPAR 2018 database (http://jaspar.genereg.net/) internal scan tool we screened these loci for the presence of putative TEAD binding sites that were evolutionary conserved among mouse, rat, dog and human genomes (49). Selected sites found in the genomic loci of *Chrna1*, *Musk* and *Dok7* genes are visualized by a sketch (Figure 10A-C), the full list of sites is presented in Supplementary Table S3. We explored the scATAC-seq dataset to examine the open chromatin regions of those three players found putative TEAD binding sites residing in open chromatin regions in human skeletal myocytes (Figure 10A-C) (79). To assess the regulatory potential of these TEAD binding sites, we cloned the genomic regions of these three synaptic players containing putative TEAD binding sites into a reporter plasmid. These genomic regions were positioned 5′ to an hsp68 minimal promoter which itself is upstream of the luciferase ORF, to investigate if TEADs could activate expression of the luciferase reporter. First, the luciferase reporter constructs were used to analyze to what degree each of the transcriptional co-activators, YAP1 or TAZ, or transcription factors, TEAD1 or TEAD4, is able to modulate luciferase activity of the reporters by potential interaction with genomic regions of the three synaptic players. Each of the expression plasmids for TEAD1, TEAD4 and constitutive active Yap5SA and TazS89A were individually transfected into cultured C2C12 muscle cells and luciferase activities were assessed in cell lysates of proliferating cells 48h after transfection. All reporters were most strongly stimulated by constitutive active TAZ in comparison with YAP1 (Figure 11A–C). TEAD1 or TEAD4 alone were not very potent in activating the reporters (Figure 11A–C). Second, each one of the three reporter plasmids was transfected into cultured C2C12 cells, differentiated to myotubes and incubated with conditioned media containing non-active (AGRN00) or active AGRN (AGRN48) for 16 hours before lysate preparation and measuring luciferase activity. As expected, all promoter fragments of synaptic players were able to significantly stimulate luciferase activity in response to active AGRN (Figure 11D-F). Third, we questioned whether mutagenesis of the M-CAT motifs of the promoters interferes with the increase of luciferase activity upon treatment of myotubes with active AGRN. All M-CAT motifs were mutagenized according to previously reported nucleobase substitutions (34,35) (Figure 10A–C). Basal luciferase activities were significantly lower whenever M-CAT motifs were mutagenized (Figure 11D–F). The incubation of transfected myotubes with active AGRN was not sufficient to stimulate the luciferase reporters with mutagenized M-CAT motifs the in the same way as with non-mutagenized ones, in comparison with inactive AGRN treatment (Figure 11D–F). Altogether, the data demonstrate that TEAD transcription factors exert direct transcriptional control over key synaptic genes, like *Chrna1*, *Musk* and *Dok7*, through evolutionary conserved regions containing TEAD binding sites, M-CAT motifs, in proximity to transcriptional start sites of these synaptic genes (Figure 10A–C).

## Discussion

Using the NMJ denervation paradigm, which is known to increase the transcription of several postsynaptic genes such as *Chrn*, and *Musk*, we identified the transcriptional co-activators of Hippo signaling, YAP1 and TAZ, and the transcription factors TEAD1 and TEAD4. All of them seem to play a role in postsynaptic gene expression. Denervation caused a significant boost in the expression of *Yap1*, *Taz*, *Tead1* and *Tead4*. This aligns with the reported elevation in *Yap1* expression after denervation and its role in attenuating denervation induced muscle atrophy (3) and promoting NMJ regeneration (33). In addition, we observed notable yet ambiguous changes in expression of TEAD target genes, namely *Cyr61*, *Ctgf* and *Ankrd1* following denervation, lending support to the occurrence of alterations in *Tead* transcriptional activity. However, transcriptional changes observed could be a consequence of a more intricate regulation including TEADs, but also other regulators, such as TGF-β signaling (80–82) or the TEAD repressors linked to the VGLL family. VGLL family members are thought to act as TEAD repressors because they bind to TEAD proteins at interfaces that physiologically interact with YAP1 or TAZ. VGLL3 and VGLL4 have both been implicated in myogenesis (24,83–85). VGLL3 has been demonstrated to suppress the expression of muscle-specific genes and is crucial for the proliferation of myoblast. However, its overexpression significantly encourages differentiation (24). VGLL4 acts a repressor of YAP1 in the proliferation phase of muscle regeneration. It also serves as a co-activator of TEAD4, which promotes *MyoG* transactivation in a YAP1-independent fashion, likely by strengthening the interaction between MYOD1 and TEAD4 (83). Consequently, it might be anticipated that *Vgll4* induction would decrease in denervated *Yap1* knockout muscles as due to the absence of YAP1 repression. However, our experiments reveal a greater expression of *Vgll4* compared to innervated *Yap1* knockout muscles. An alternative explanation could be an upregulation of *Taz* expression in the *Yap1* knockout muscles. The transcription of Vgll3 and Vgll4 was examined in adult control mice, revealing their downregulation following denervation. Similarly, VGLLs were downregulated in neonatal single and double knockout muscles, which could suggest their expression in synaptic nuclei. Notably, *Yap1*, *Taz*, *Tead1* and *Tead4* were expressed in differentiated muscle cells and induced by neural AGRN. As a result, we examined the effects of *Yap1, Taz*, *Tead1* and *Tead4* deficiency on differentiated primary cultured muscle cells and observed reduced synaptic gene expression and impaired AGRN-induced CHRN clustering. Since TEADs can directly regulate muscle-related gene expression by binding to M-CAT sequences found upstream of genes encoding CHRN subunits in chicks and rats (77,78), we hypothesized that TEADs bind directly to these M-CATs and regulate key synaptic gene expression in mice.

Through *in silico* screening of previously reported TEAD ChIP-Seq data using C2C12 cells (47), here we show that in differentiated C2C12 cells TEAD4 but not TEAD1 occupied regions are located in the vicinity of the transcription start site of postsynaptic genes, like *Chrna1*, *Chrng*, *Musk*, *Dok7*, *Utrn* and *Dtna* (Supplementary Tables S2, S3). Further, we found that these genes are characterized by possessing evolutionarily conserved putative TEAD binding sites in their promoter regions, which are located in open chromatin regions in human skeletal myocytes (Figure 10A–C, Supplementary Figures 3 and 4). Consistent with the enhanced expression of synaptic genes upon of muscle cell differentiation (69,86,87), analysis of luciferase reporter constructs with exemplary three of these genes, *Chrna1*, *Musk*, and *Dok7*, demonstrated that these TEAD-occupied genomic regions were sufficient to induce TEAD-mediated transcription, predominantly by TAZ, in proliferating C2C12 myoblasts. In differentiated C2C12 myotubes, neural AGRN stimulated postsynaptic gene expression by these TEAD occupied regions, but failed to do so when these regions were mutagenized. Neural AGRN activity was necessary and sufficient to raise TEAD4 levels in cultured myotubes. TEAD4 may enhance AGRN/MUSK/LRP4 induced synaptic transcription through stimulation of *Musk* expression, as well as directly regulate the expression of other genes, such as *Chrna1* and *Dok7*. While our findings may be limited to the analyzed postsynaptic targets, they suggest the crucial role of YAP1, TAZ, and TEAD4 as important neuromuscular regulators. All investigated target genes, *Chrna1*, *Chrng*, *Musk*, *Dok7*, *Utrn* and *Dtna*, are essential for the formation of postsynaptic CHRN clusters (26). Based on TEAD1 ChIP-Seq data in C2C12 cells (47), TEAD1 was found to not occupy any proximal regions related to the synaptic genes analyzed, irrespective of the differentiation stage of the C2C12 cells. Our analysis of transcriptional changes in *Tead1* knockout cells showed that of the four synaptic genes examined, only *Chrng* was significantly downregulated and that the transcription of TEAD target genes differed from that in *Tead4* knockout cells. Of note, transcript levels of postsynaptic genes *Chrnb* and *Rapsn* are significantly reduced in double knockout muscles and their genomic regions possess M-CAT motifs in evolutionary conserved and open chromatin genomic regions (data not shown), but these regions have not been previously identified by ChIP-Seq data (47). Interestingly, *Ctgf* was strongly downregulated in *Tead1* and upregulated in *Tead4* knockout cultured muscle cells. It has previously been reported that Ctgf knockout mouse embryos exhibit impaired NMJ transmission (88). CTGF appears to interact with LRP4 to facilitate the clustering of CHRNs at NMJs and the maturation of nerve terminals (88). Further exciting investigations remain to determine whether and to what extent CTGF also impairs clustering of CHRNs in adult skeletal muscles. In theory, both TEAD1 and TEAD4 can recognize the same M-CAT-like DNA sequences, but the resulting transcription may differ due to tissue and interaction specificity (76). Considering the similar expression patterns and neural AGRN-induced CHRN clustering deficits in CRISPR knockout myotubes for *Tead1* and *Tead4*, both transcription factors are involved in regulating of CHRN clustering and synaptic gene expression, although they may occupy different gene sets under physiological conditions, similar to their reported requirement for muscle cell differentiation (47). The corresponding transcriptional activator of TEAD-mediated synaptic gene expression remains to be determined. Both, *Yap1* and *Taz* transcription increased after denervation and, together with our data and a previous report (3), may indicate their involvement at different developmental stages. Previous structural data suggest that YAP1 and TAZ bind to the same site on TEADs. Importantly, it was found that secondary structural elements of their TEAD binding site do not contribute equally to the overall affinity, and critical interactions with the TEAD occur through different residues (85). We also observed increased *Yap1* but also *Taz* transcription in myotubes treated with neural AGRN. In the context of liver cells, *Yap1* expression is directly regulated by the Ets family transcription factor GABP (89), which is also an important regulator of synaptic gene expression (90), and there YAP1 has been linked to AGRN/MUSK signaling (91,92). A recent study of the role of YAP1 at the NMJ, identified impaired CTNNB1 signaling downstream of YAP1 as a possible mechanism for pre- and postsynaptic deficits in muscle-specific *Yap1* knockout mice (33). However, the same report found that the expression of synaptic genes encoding *Musk* and *Chrnb* was not affected in *Yap1* knockout muscles (33). In this context, it is possible that the structurally and functionally similar TAZ takes over the role of YAP1 in regulating TEAD-mediated synaptic gene transcription in non-physiological context. A direct comparison of the neuromuscular phenotype of conditional muscle-specific *Yap1* and *Taz* single knockouts or *Yap1*/*Taz* double knockouts would help to understand their different roles. Our data using such mouse models suggest that TAZ plays a more important role than YAP1 in regulating synaptic gene expression. While TAZ appears to be more important for physiological synaptic gene transcription at NMJs, YAP1 may be more involved under pathological conditions. Consistently, YAP1 levels are reduced in muscle biopsies from obese, insulin-resistant humans and mice (93).

Recently, two roles for CTNNB1 have been proposed: a TCF/LEF-independent nuclear function that co-ordinates myogenic genes together with MyoD1; and an α-catenin-dependent membrane function that helps control cell–cell interactions (94). Furthermore, the existence of transcriptional activity of CTNNB1 in the absence of TCF/LEF factors has been demonstrated (95). In myogenesis, negative feedback regulates myotube formation by increasing CTNNB1-dependent *Axin2* expression and YAP1/TAZ-TEAD signaling activity in response to canonical Wnts (25). Previous reports indicate a similar effect of both Wnt and YAP1/TAZ-TEAD signaling pathways in regulating CHRN clustering. On the one hand, canonical and non-canonical Wnt pathways play opposing roles at the NMJ but complement each other in regulating the assembly and maintenance of the postsynaptic apparatus through anterograde and retrograde signaling (96). On the other hand, YAP1 cooperates with AGRN/MUSK/LRP4 signaling in NMJ formation and regeneration (33) and counteracts neurogenic atrophy in denervated muscles (3). Our results indicate that TEADs directly regulate synaptic gene expression and affect AGRN induced CHRN clustering. The increase in *Yap1*/*Taz*/*Tead* expression and activity, observed shortly after denervation, may represent a physiological response to promote re-innervation and synaptic gene expression, and counteract muscle atrophy caused by denervation induced muscle atrophy.

## Supplementary Material

gkad1124_supplemental_file

## Data Availability

The data underlying this article are available in the Gene Expression Omnibus at https://www.ncbi.nlm.nih.gov/geo/ and can be accessed under accession code GSE217576. All further data generated or analyzed during this study are included in this published article and its supplementary file.
