# Supplementary material for: The YAP1/TAZ-TEAD transcriptional network regulates gene expression at neuromuscular junctions in skeletal muscle fibers
Source: Nucleic Acids Res. 2023 Dec 4;52(2):600–24. doi: 10.1093/nar/gkad1124 (PMC10810223; doi:10.1093/nar/gkad1124)
Supplement: gkad1124_supplemental_file [file gkad1124_supplemental_file.pdf]

The YAP1/TAZ-TEAD transcriptional network regulates gene expression at neuromuscular junctions in skeletal muscle fibers

Short title: YAP1/TAZ-TEAD at NMJ

Authors

Lea Geßler [1]#, Danyil Huraskin [1]#, Yongzhi Jian [1], Nane Eiber [1], Zhaoyong Hu [2], Tomasz J. Prószyński [3] and Said Hashemolhosseini [1, 4] *

Affiliations

[1] Institute of Biochemistry, Medical Faculty, Friedrich-Alexander-University of Erlangen-Nürnberg, 91054 Erlangen, Germany

[2] Nephrology Division, Department of Medicine, Baylor College of Medicine, Houston, TX, USA

[3] Łukasiewicz Research Network-PORT Polish Center for Technology Development, Wrocław, Poland

[4] Muscle Research Center, Friedrich-Alexander-University of Erlangen-Nürnberg, 91054 Erlangen, Germany

# equal contribution

Corresponding author *

Said Hashemolhosseini, Institut für Biochemie, Friedrich-Alexander-Universität Erlangen-Nürnberg, Fahrstrasse 17, 91054 Erlangen, Germany, Phone: +49 9131 85 24634, Fax: +49 9131 85 22484, E-mail: said.hashemolhosseini@fau.de

Keywords

YAP1, TAZ, TEAD, synaptic gene expression, neuromuscular junction

Key Points

1. Neuromuscular junctions (NMJs) and the organization of synaptic nuclei are impaired when *Yap1* and/or *Taz* are knocked out specifically in muscle fibers.

2. Muscle-specific double knockout mice do not survive beyond birth, likely due to almost absent and severely disorganized NMJs.

3. *In vitro* and *in vivo* experiments demonstrate that YAP1, TAZ, TEAD1 and TEAD4 differentially regulate transcription of synaptic genes at NMJs.

SUPPLEMENTARY DATA

Supplementary figure 1: 3D images of BTX and DAPI stained NMJs in soleus single fibers.

(A-C) Representative NMJ images are shown for each genotype (control, *Yap1* knockout and *Taz* knockout diaphragm muscles). CHRN clusters are stained with BTX, while myonuclei underneath pretzel-shaped NMJs are visualized by the DAPI stain. Note, any additional nuclei observed at NMJs of *Taz* knockout muscles are presumed to belong to the muscle fibers as they are located within the same z-layer as the BTX stain.

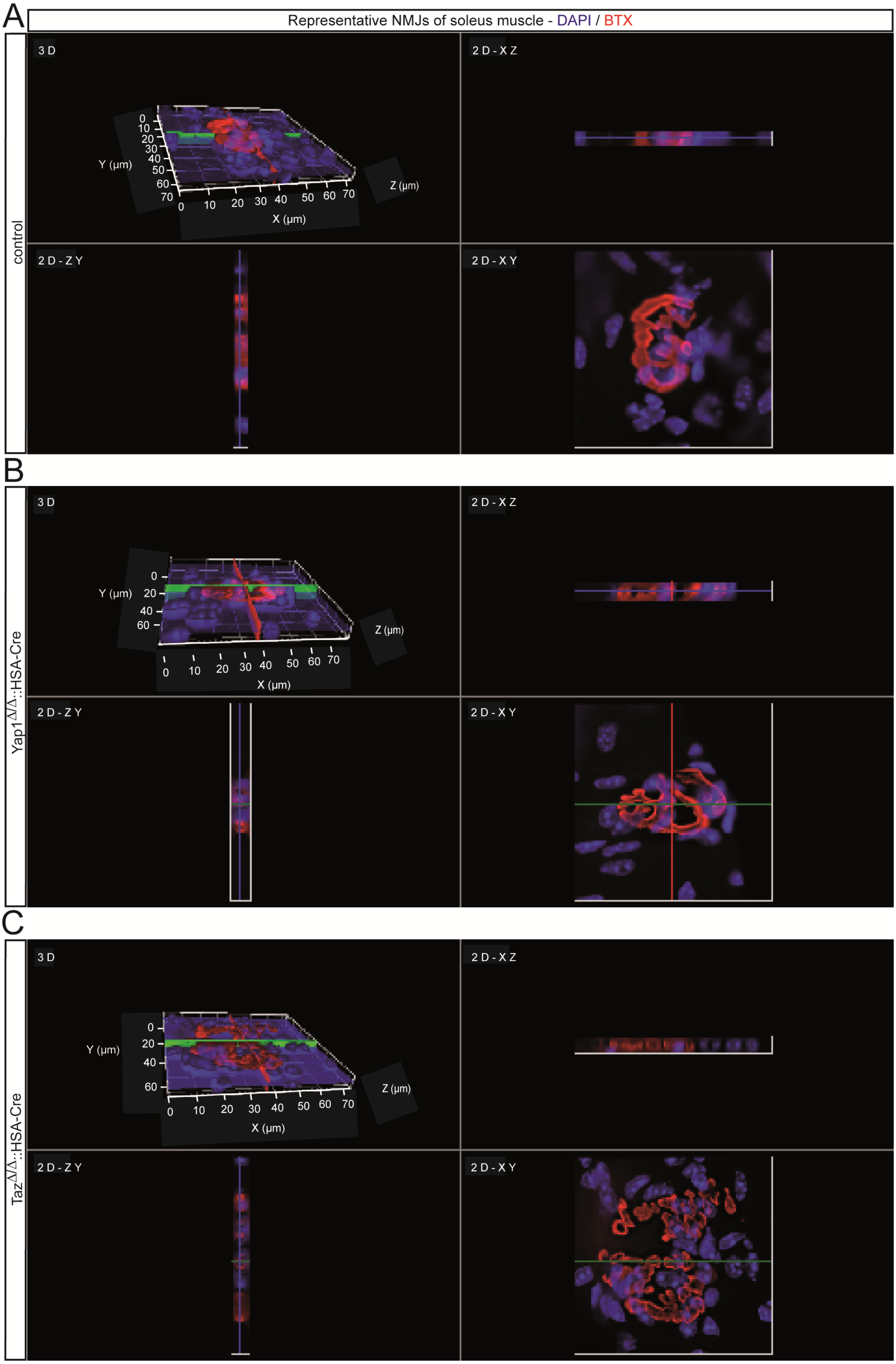
A
Representative NMJs of soleus muscle - DAPI / BTX
control
3 D
2 D - X Z
2 D - Z Y
2 D - X Y
Y (μm)
X (μm)
Z (μm)
B
Yap1Δ/Δ::HSA-Cre
C
TazΔ/Δ::HSA-Cre

Supplementary figure 2: Generation and validation of Tead1 and Tead4 knockout primary muscle cells by CRISPR/Cas9 genome editing.

(A) For CRISPR/Cas9-mediated knockout of the genes of interest optimal 20bp guide sequences (blue) adjacent to 3-mer PAM sequence NGG (green) were chosen in one of the early coding exons to favor frameshift mutations that would result in a premature stop and nonsense mediated decay of target peptides. The actual indels in any of the clones used in this study, as confirmed by genomic sequencing, are shown in red for each allele and compared against the wild type genomic sequence. The resulting protein variants are designated in parentheses according to recommendations of the Human Genome Variation Society. For example, the *Tead1* knockout clone contains a 2bp deletion on one copy of the gene, resulting in a frameshift with Alanine 16 as the first amino acid substituted to Glycine and a stop-codon 7 amino acids later. The second allele contains a 1bp deletion at the same position, resulting in a frameshift starting with substitution of Alanine 16 to Arginine and a stop codon 42 amino acids later. The genomic coordinates of the presented sequences are based on the Mouse July 2007 (NCBI37/mm9) genome assembly. (B) According to Western blot analysis the target protein is undetectable in the protein lysates of the respective knockout clones. (C) The absence of the target protein was further confirmed by immunofluorescence microscopy. N≥3 set of cells.

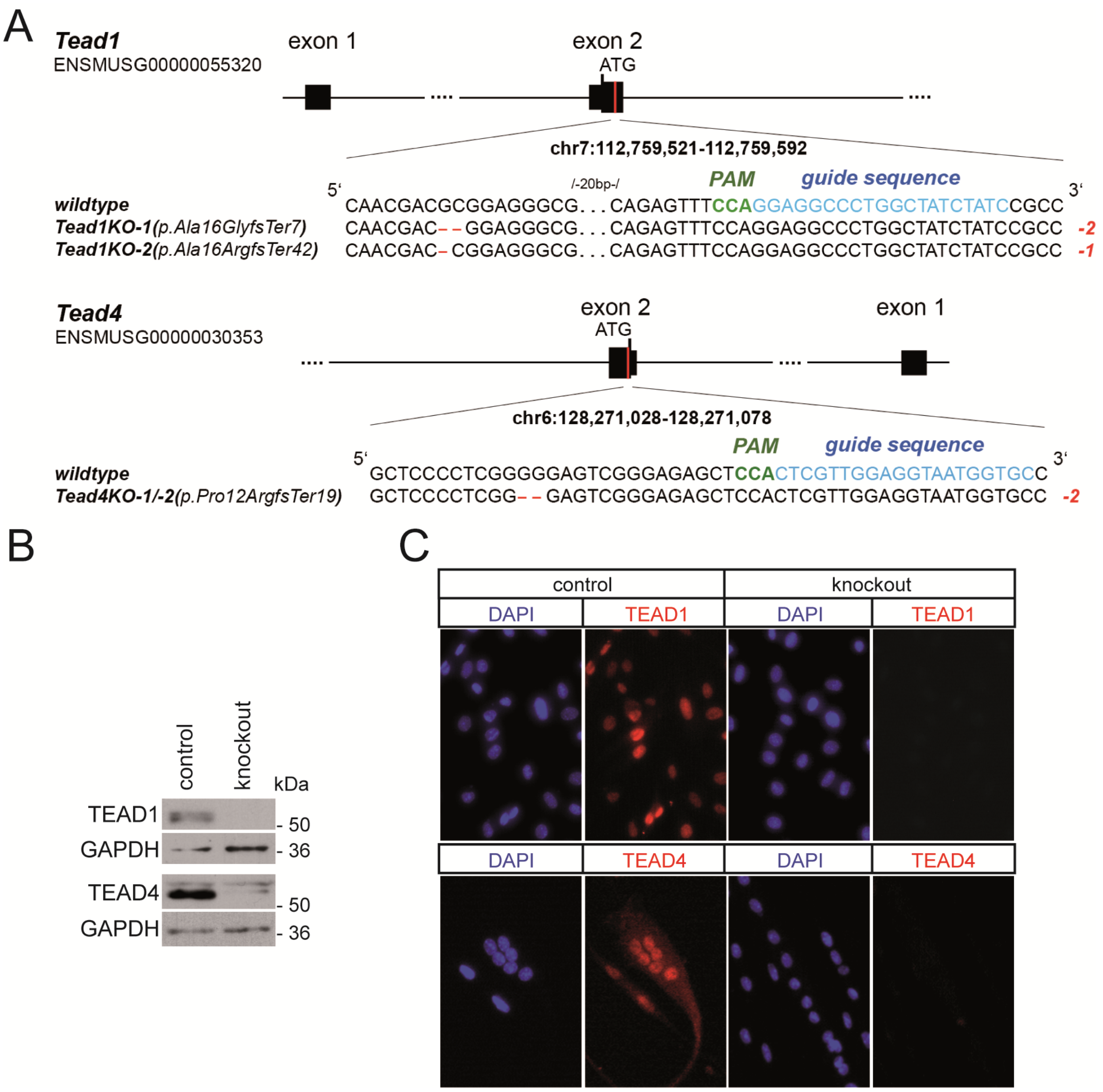

A
Tead1
ENSMUSG00000055320
exon 1
exon 2
ATG
chr7:112,759,521-112,759,592
PAM
guide sequence
/-20bp-/
wildtype
Tead1KO-1(p.Ala16GlyfsTer7)
Tead1KO-2(p.Ala16ArgfsTer42)
CAACGACGCGGAGGGCG. . . CAGAGTTTCCAGGAGGCCCTGGCTATCTATCCGCC
CAACGAC– –GGAGGGCG. . . CAGAGTTTCCAGGAGGCCCTGGCTATCTATCCGCC -2
CAACGAC–CGGAGGGCG. . . CAGAGTTTCCAGGAGGCCCTGGCTATCTATCCGCC -1
Tead4
ENSMUSG00000030353
exon 2
ATG
exon 1
chr6:128,271,028-128,271,078
PAM
guide sequence
wildtype
Tead4KO-1/-2(p.Pro12ArgfsTer19)
GCTCCCCTCGGGGGAGTCGGGAGAGCTCCACTCGTTGGAGGTAATGGTGCC
GCTCCCCTCGG– –GAGTCGGGAGAGCTCCACTCGTTGGAGGTAATGGTGCC -2
B
control
knockout
kDa
TEAD1
GAPDH
TEAD4
GAPDH
- 50
- 36
- 50
- 36
C
control
knockout
DAPI
TEAD1
DAPI
TEAD1
DAPI
TEAD4
DAPI
TEAD4

Supplementary figure 3, 4: TEADs bind to M-CAT motifs in evolutionarily conserved and open chromatin regions of postsynaptic key genes, such as Chrng, Utrn and Dtna.

A search for putative M-CAT motifs for TEAD transcription factors was performed in evolutionary conserved regions of synaptic genes previously occupied by TEAD4 in ChIP-Seq experiments in differentiated C2C12 muscle cells (1). Several putative M-CAT binding sites that were highly conserved among mammalian species were identified. Three representative ones (Chrna1, Musk, and Dok7) are shown in the manuscript (Fig. 10, 11). Additional genes were identified and are shown by this supplementary figure, such as *Chrng* (suppl. fig. 3A), *Utrn* (suppl. fig. 3B), and *Dtna* (suppl. fig. 4). The genomic loci are displayed indicating highly conserved areas, such as gene exons or possible regulatory regions, with PhyloP conserved score peaks. TEAD4-occupied sites are presented as bright green rectangles, while putative TEAD4 binding sites are labeled in red and presented in boxes with multiple alignments of respective genomic sequences from various mammalian species. The search for TEAD4 putative binding sites was performed with the JASPAR 2018 Scan function (TEAD4 matrix profile ID: MA0809.1) and the default relative score profile threshold of 80%. The relative scores of each site are specified under the multiple alignment boxes. Further, scATAC-seq dataset was explored to examine open chromatin regions of above-mentioned genes. Notably, the identified M-CAT motifs are located in open chromatin regions of human skeletal myocytes.

Supplementary figure 3

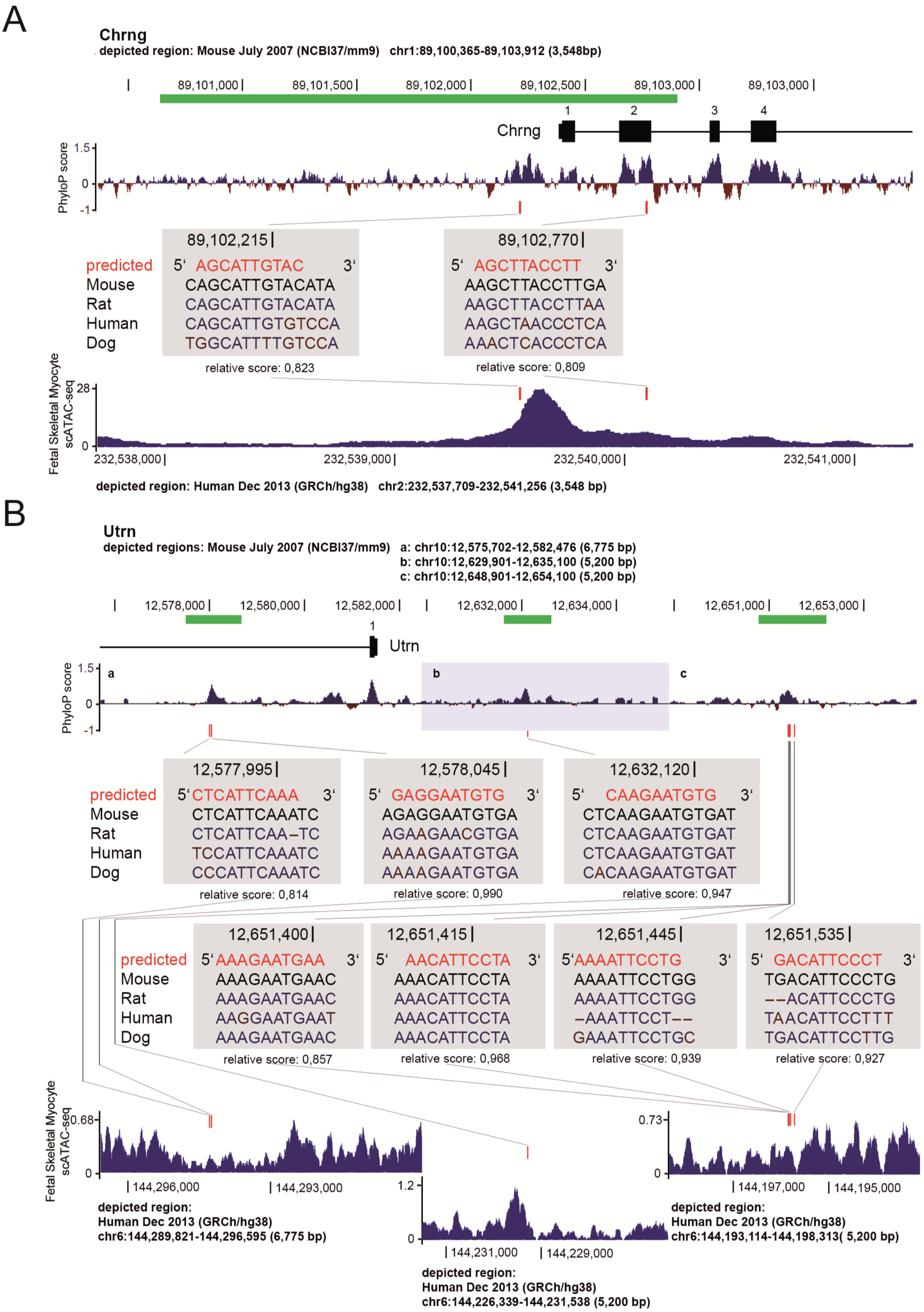

Supplementary figure 4

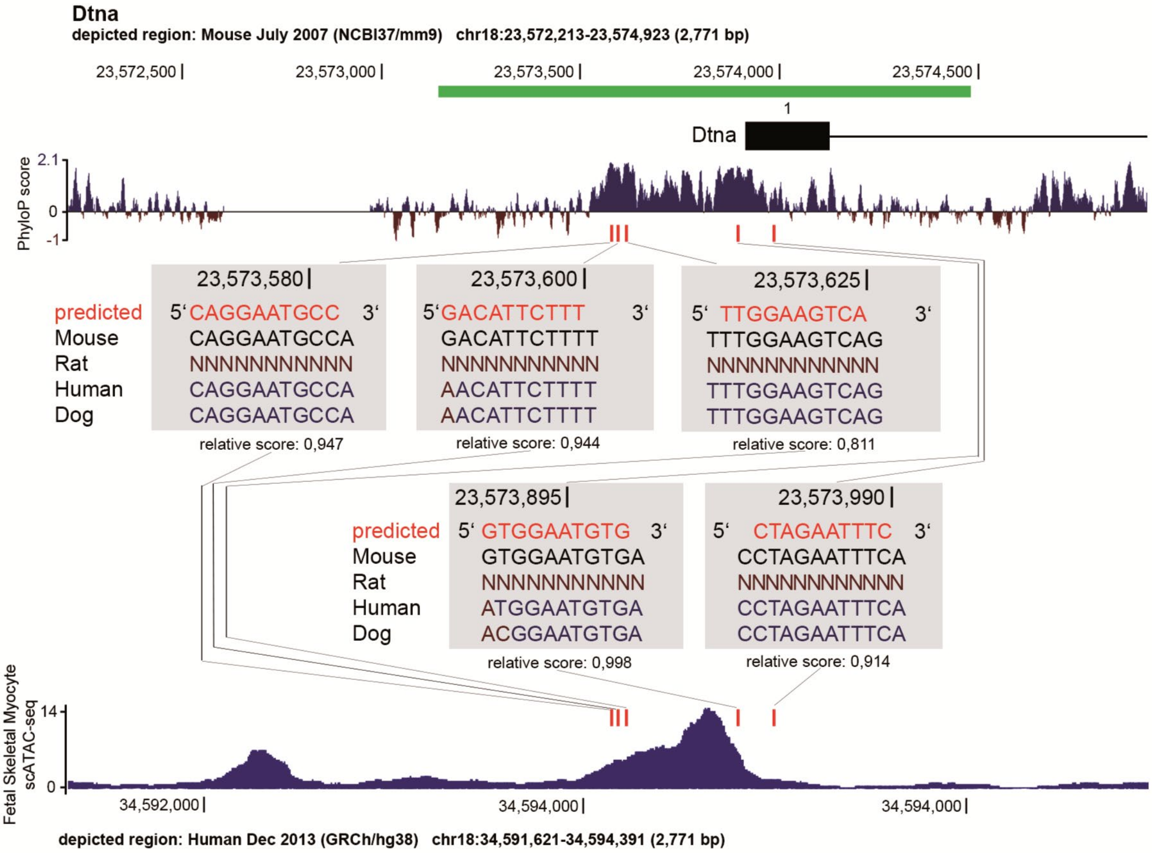

Supplementary Table S1

Tabular presentation of oligonucleotide sequences.

Genomic target	Orientation	Sequence
Tead1 target sequence for cloning into pX330	forward	CACCGCCGATTGACAACGACGCGG
	reverse	AAACCCGCGTCGTTGTCAATCGGC
Tead4 target sequence for cloning into pX330	forward	CACCGAGCTCTCCCGACTCCCCCG
	reverse	AAACCGGGGGAGTCGGGAGAGCTC
pX330 sequencing		GGCCTATTTCCCATGATTCCTTC
Tead1 amplification and sequencing of CRISPR/Cas9 target site	forward	CAGGCTTCGGCTTGGAAAAC
	reverse	TGGAGGCCCTGAAAGGAATG
Tead4 amplification and sequencing of CRISPR/Cas9 target site	forward	GTCAGGATAATTTTGCGGCGG
	reverse	GCCTTGTTTTGGCTCTAACAG
Tead1 qPCR and In situ riboprobes	forward	TCAAGCCGCCATTAAGGTGT
	reverse	GGCTTGACGTCTTGTGAGGA
Tead2 qPCR	forward	GATCCTGCTTGGGGTTTGCC
	reverse	GTTCCGGCCATACATCTTGC
Tead3 qPCR	forward	AAAACAGGTGTCCAGCCACA
	reverse	GGGCGAGGGACTCATAACTG
Tead4 qPCR and In situ riboprobes	forward	ATGCAGAGGGTGTATGGAGC
	reverse	TTCTTAGCTGCCTGGTCCTTG
Musk qPCR	forward	GCCTTGGTTGAAGAAGTAGC
	reverse	CTTGATCCAGGACACAGATG
Dok7 qPCR	forward	GAATTCGGTTCTCTGCTCAGTCTG
	reverse	CCAAGTCCATGTAGTGCAGCTG
Dtna qPCR	forward	ATTAAGTGGGCGCCTTCCTC
	reverse	AGTCCCCACGGGTCAAATTC
Chrna1 qPCR	forward	ACGCTGAGCATCTCTGTCTT
	reverse	TTGGACTCCTGGTCTGACTT
Chrnb1 qPCR	forward	ATAGGTACCCAGCATACCATATCAGAACG
	reverse	ATACTCGAGCTGAACGGATCAAGAACCAC
Chrng qPCR	forward	GGTCAATGTCAGCCTGAAGC
	reverse	GCACATGCATCCGTAACAGC
Chrnd qPCR	forward	ATGAGGAACAAAGGCTGATCCA
	reverse	ACAGTGATGTTCCCGAAGTCGT
Ctnnb1 qPCR	forward	TCTGGAATCCATTCTGGTGC
	reverse	CTCATCTAGCGTCTCAGGGA
Yap1 qPCR	forward	TTCGGCAGGCAATACGGAAT
	reverse	GTTGAGGAAGTCGTCTGGGG
Wwtr1 qPCR	forward	GTTCCGGGGATAAAGATGAATCCG
	reverse	GAAGTGATGGACGGGTGGAG
Rpl8 qPCR	forward	GTTCGTGTACTGCGGCAAGA
	reverse	ACAGGATTCATGGCCACACC
Cyr61 qPCR	forward	AAGAGGCTTCCTGTCTTTGGC
	reverse	ATCGGAACCGCATCTTCACA
Ctgf qPCR	forward	CTAGCTGCCTACCGACTGGAA
	reverse	CAAACTTGACAGGCTTGGCG
Ankrd1 qPCR	forward	TGGAGGAAACGCAGATGTCC
	reverse	TCCCAGCACAGTTCTTGACC
Vgll3 qPCR	forward	GGATTCCTGCTCCCCAGT
	reverse	TTGTCCTGATGCTGAAGACCT

Vgll4 qPCR	forward	TGTGAAAACGACCACGTCTC
	reverse	GCAGTCTCCGTTGACAGTCTT
Myog qPCR	forward	AGTGAATGCAACTCCCACAG
	reverse	CTGGGAAGGCAACAGACATA
Slit1 qPCR	forward	GAACTCAACGGCAACAACATC
	reverse	TCAGGCAACACTTGTAGCTGG
Slit2 qPCR	forward	GGCAGACACTGTCCCTATCG
	reverse	ATCTATCTTCGTGATCCTCGTGA
Rapsn qPCR	forward	ATAGGTACCCAAGAATCAGCAGCAAGGCGAA
	reverse	ATACTCGAGAAGGCTGGTTCAGCCCCTGT
Utrn qPCR	forward	ATAGGTACCGTGAGCTATATCCCAGAATC
	reverse	ATACTCGAGTACTCTCGATTCTATCTCCA
Dok7_138-137 MCAT 920	forward	AGACGAGCGCTGGCCTTGGCCCAA
	reverse	TAGAGCAGGACGTGGCCCCTCCCTG
Dok7_138-137 MCAT 070	forward	GATGCCCACACACTGAAGGCCCCTG
	reverse	TTAGATGGGGCTCTGTGGCCAGC
AChRa_11-10 MCAT 475	forward	AGAGGTTAACATTTCACACCTCCTAG
	reverse	GAGCTTCACTTGTGTTCCTCAAATAAC
AChRa_11-10 MCAT 530	forward	TTACTCCTAGACCAAATTGAAACTAATTAAAAG
	reverse	GACCCTGTTGACACTCTGACAAAAAC
AChRa_6-7 MCAT 480	forward	ACGGAGCAGAGAGTAGGTCAAGGGC
	reverse	CTTAGGTCCATCACAACAGCTGAAAC
MuSK_170-171 MCAT 735	forward	AGACGAAGGCACTGGAAAAAAAAAAAAAG
	reverse	TAGACCGGAAATAACACCACCTAAG
MuSK_170-171 MCAT 780	forward	ACGTAAGTTGCTTTCCCCCTTAGG
	reverse	CTGAGACAAACACCTGTTGCAGCAG
MuSK_170-171 MCAT 810	forward	GATGGCTGCTGCAACAGGTGTTTGTC
	reverse	TTAGCCACAGAGCAGTGGTTTCTGGC
AChRa-6_7	forward	GGTACCGGTGAAACACTACATTGCCA
	reverse	CTCGAGTGTAGTGTTTGTAAACTCATGCA
AChRa-10_11	forward	ATAGGTACCATAGTCCCTGGATCAGGCAC
	reverse	ATACTCGAGTCTGTTTCTGGGTGAGGTAG
AChRa-12_13	forward	ATAGGTACCGCTGGCCTTGGAAGCCAGCT
	reverse	ATACTCGAGACTGCCTGACAGAGATGAGG
Dok7-137_138	forward	ATAGGTACCCGAGTCCTCCATTGGCCGAT
	reverse	ATACTCGAGCCGAGAGTCCTTGCCACAGC
MuSK-170_171	forward	ATAGGTACCGAGAAGCATATACAGACTGAT TGT
	reverse	ATACTCGAGATAGGTCCAAGCAGCCAGGT

Supplementary Table S2

Summary of previously reported TEAD1- and TEAD4 occupied sites found in the vicinity of synaptic genes *Chrna1*, *Chrng*, *Musk*, *Dok7* and *Utrn* in C2C12 cells at day 0 and day 6 of differentiation (1).

**TEAD1 - day 0**			
gene	*chromosome*	*start*	*end*
-	-	-	-
**TEAD1 - day 6**			
gene	*chromosome*	*start*	*end*
-	-	-	-
**TEAD4 - day 0**			
gene	*chromosome*	*start*	*end*
Utrn	chr10	12414469	12414921
	chr10	12523559	12524217
	chr10	12577638	12578426
	chr10	12631909	12632422
**TEAD4 - day 6**			
gene	*chromosome*	*start*	*end*
Chrna1	chr2	73416829	73419463
	chr2	73424990	73425774
Chrng	chr1	89097639	89098621
	chr1	89100634	89102900
Musk	chr4	58299279	58300387
	chr4	58414670	58415456
	chr4	58425815	58427149
	chr4	58429817	58430836
Dok7	chr5	35397473	35398478
Utrn	chr10	12211434	12212240
	chr10	12292305	12293128
	chr10	12414426	12415324
	chr10	12523192	12524140
	chr10	12530403	12531951
	chr10	12577499	12578667
	chr10	12631629	12632616
	chr10	12650779	12652206

Supplementary Table S3

Full list of identified putative TEAD4 binding sites in TEAD4-occupied regions in the vicinity of synaptic genes *Chrna1*, *Chrng*, *Musk*, *Dok7*, *Utrn*, and *Dtna*, in C2C12 cells according to ChIP-Seq data in (1). Genomic coordinates refer to Mouse July 2007 (NCBI37/mm9) genome assembly. Note, all sites, but *Utrn* sites 4-6, are located in regions occupied by TEAD4 exclusively in differentiated C2C12 cells. *Utrn* sites 4-6 overlap with a region occupied by TEAD4 in both undifferentiated and differentiated C2C12 cells (see Suppl. Table S2). *Chrng* #2 is incorporated in suppl. fig. 3 albeit being part of exon2 and intragenic, while *Utrn* #1 is not part of suppl. fig. 3 because it is too much intragenic (1).

**Full list of putative TEAD4 binding sites**							
gene	*#*	*strand*	*sequence*	*chromosome*	*start*	*stop*	*score*
Chrna1	1	+	CACATTCCAG	chr2	73417477	73417486	0,987
	2	-	AACATTTCAC	chr2	73425458	73425467	0,880
	3	+	AACATTGTTC	chr2	73425469	73425478	0,832
	4	-	AATATTCCTA	chr2	73425525	73425534	0,855
	5	+	AATATTCCAC	chr2	73425529	73425538	0,871
Chrng	1	+	AGCATTGTAC	chr1	89102210	89102219	0,823
	2	+	AGCTTACCTT	chr1	89102762	89102771	0.809
Musk	1	-	TACATTCCAA	chr4	58299729	58299738	0,975
	2	-	TGCTTTCCCC	chr4	58299760	58299769	0,804
	3	-	CACTTTCTTA	chr4	58299773	58299782	0,845
	4	-	GACATTCTTG	chr4	58299803	58299812	0,931
	5	-	TGCATTCTTG	chr4	58426480	58426489	0,914
	6	+	TGCATACCAG	chr4	58426486	58426495	0,908
	7	+	AAAATTCCTT	chr4	58426498	58426507	0,952
	8	+	TACATTCTTT	chr4	58426507	58426516	0,944
Dok7	1	-	TGCATTCCAA	chr5	35397914	35397923	0,958
	2	-	TTCATACTAC	chr5	35398060	35398069	0,816
Utrn	1	-	GAAATTCCAG	chr10	12292949	12292958	0,946
	2	+	CTCATTCAAA	chr10	12577987	12577996	0,814
	3	-	CACATTCCTC	chr10	12578037	12578046	0,990
	4	-	CACATTCTTG	chr10	12632114	12632123	0,947
	5	-	TTCATTCTTT	chr10	12651393	12651402	0,857
	6	+	AACATTCCTA	chr10	12651410	12651419	0,968
	7	+	AAAATTCCTG	chr10	12651439	12651448	0,939
	8	+	GACATTCCCT	chr10	12651530	12651539	0,927
Dtna	1	-	CACATTCCAC	chr18	23573891	23573900	0.998
	2	-	GGCATTCCTG	chr18	23573574	23573583	0.947
	3	+	GACATTCTTT	chr18	23573592	23573601	0.944
	4	-	GAAATTCTAG	chr18	23573982	23573991	0.914
	5	-	TGACTTCCAA	chr18	23573617	23573626	0.811

MATERIALS AND METHODS

Generation of CRISPR/Cas9 knockout cells

For CRISPR/Cas9 mediated gene editing guide sequences (spacers) were designed with the online tool E-CRISP (2) (suppl. table S1). Search parameters were set to medium stringency and sequences were chosen, that were exonal and closest to the ATG in 3' direction. 20bp long complementary oligonucleotide pairs with overhangs were cloned into the pX330-U6-Chimeric_BB-CBh-hSpCas9 vector from Feng Zhang Lab (Addgene plasmid # 42230). Cloning procedure was based on a published protocol (3) and available online at: *https://media.addgene.org/cms/filer_public/e6/ 5a/e65a9ef8-c8ac-4f88-98da-3b7d7960394c/zhang-lab-general-cloning-protocol.pdf* . Plasmids were transformed in NEB 5-α *E.coli* bacteria (New England Biolabs, C2987), extracted from bacteria by alkaline lysis with the Nucleobond PC100 Midiprep Kit (Macherey-Nagel, 740573) and verified by restriction digestion and sequencing.

For CRISPR/Cas9-mediated generation of knockout cells, guide sequences were designed to target the coding sequence closest to the start codon of the gene of interest to hit as many splice variants as possible, and were cloned into pX330-U6-Chimeric_BB-CBh-hSpCas9 vector (3). For *Tead1* or *Tead4* knockout the respective sequences were located in exon2 (suppl. fig. 2A). 24h after co-transfection of the respective vectors and a GFP expressing plasmid into purified primary wild type muscle satellite cells with a low passage number (less than 3), single cells were detached and single-cell FACS sorted onto Matrigel-coated 96 Well plates containing growth medium and clonally expanded. After clonal expansion, several independent correct knockouts for *Tead1* and *Tead4* were identified by absence of protein of interest by immunofluorescence microscopy and Western Blot (suppl. fig. 2B, C). Clones of each gene knockout were sequenced to confirm and identify the genomic bi-allelic mutations (suppl. fig. 2A). For this, a region with about 100-150 bps flanking each side of the target site was amplified and the PCR products were sequenced with one of the primers (suppl. table S1). The Degenerate Sequence Decoding strategy (4) was employed to decode sequences of each allele from overlapping peaks of sequencing chromatograms. Clones with undetectable protein of interest consistently featured indels in the vicinity of the target sequence. Only clones with frameshift mutations causing premature stops on both alleles were used for further studies.

REFERENCES

1. Joshi, S., Davidson, G., Le Gras, S., Watanabe, S., Braun, T., Mengus, G. and Davidson, I. (2017) TEAD transcription factors are required for normal primary myoblast differentiation in vitro and muscle regeneration in vivo. *PLoS genetics*, **13**, e1006600.
2. Heigwer, F., Kerr, G. and Boutros, M. (2014) E-CRISP: fast CRISPR target site identification. *Nature methods*, **11**, 122-123.
3. Cong, L., Ran, F.A., Cox, D., Lin, S., Barretto, R., Habib, N., Hsu, P.D., Wu, X., Jiang, W., Marraffini, L.A. *et al.* (2013) Multiplex genome engineering using CRISPR/Cas systems. *Science*, **339**, 819-823.
4. Ma, X., Chen, L., Zhu, Q., Chen, Y. and Liu, Y.G. (2015) Rapid Decoding of Sequence-Specific Nuclease-Induced Heterozygous and Biallelic Mutations by Direct Sequencing of PCR Products. *Mol Plant*, **8**, 1285-1287.
